# Strigolactones GR-24 and Nijmegen Applications Result in Reduced Susceptibility of Tobacco and Grapevine Plantlets to *Botrytis cinerea* Infection

**DOI:** 10.3390/plants12183202

**Published:** 2023-09-07

**Authors:** Dominic Vogel, Paul Hills, John P. Moore

**Affiliations:** 1 South African Grape and Wine Research Institute, Department of Viticulture and Oenology, Faculty of AgriSciences, Stellenbosch University, Stellenbosch 7600, South Africa; dominicvogel1@gmail.com; 2 Institute for Plant Biotechnology, Department of Genetics, Faculty of AgriSciences, Stellenbosch University, Stellenbosch 7602, South Africa; phills@sun.ac.za

**Keywords:** *Botrytis cinerea*, plant fitness, reactive oxygen species, strigolactones, hyphal branching

## Abstract

Priming agents are plant defence-inducing compounds which can prompt a state of protection but may also aid in plant growth and interactions with beneficial microbes. The synthetic strigolactones (±)-GR24 and Nijmegen-1 were evaluated as potential priming agents for induced resistance against *Botrytis cinerea* in tobacco and grapevine plants. The growth and stress response profiles of *B. cinerea* to strigolactones were also investigated. Soil drench treatment with strigolactones induced resistance in greenhouse-grown tobacco plants and restricted lesion development. The mode of action appeared to function by priming redox-associated compounds to produce an anti-oxidant protective response for limiting the infection. The results obtained in the in vitro assays mirrored that of the greenhouse-grown plants. Exposure of *B. cinerea* to the strigolactones resulted in increased hyphal branching, with (±)-GR24 stimulating a stronger effect than Nijmegen-1 by affecting colony diameter and radial growth. An oxidative stress response was observed, with *B. cinerea* exhibiting increased ROS and SOD levels when grown with strigolactones. This study identified the application of strigolactones as potential priming agents to induce disease resistance in both tobacco and grapevine plants. In addition, strigolactones may alter the ROS homeostasis of *B. cinerea*, resulting in both morphological and physiological changes, thereby reducing virulence.

## 1. Introduction

Plants can identify numerous environmental signals that allow them to react to their environment [1]. Priming stimuli can include a wide range of physical, biological, or chemical environmental signals, which can prompt the induction of priming by acting as warning signals. These stimuli induce low-metabolic-cost changes in the plant, including accumulating various plant defence-associated metabolites [2]. The accumulation of these metabolites triggers a physiological state that enhances induced defences and improves the plant’s performance upon challenge [3]. This is the case, for example, for some beneficial microbes, including plant-growth-promoting rhizobacteria and fungi, which can induce positive effects in the interaction between microbes and plant hosts [4].

Priming can be defined by its overall positive cost–benefit balance in times of stress to the plant [5]. Although the benefits of priming have been well interpreted, surprisingly, few studies have evaluated the fitness effects of priming agents [6]. During this priming time, the defence responses triggered are unclear and are only often slightly and transiently triggered by the priming stimulus and return to nearly basal levels until the stress is induced [7]. Although a stronger and quicker defence response is associated with the primed state, the heightening of inducible defence traits does not always provide benefit. For example, negative hormonal crosstalk has been described for induced defences against herbivores and necrotrophic pathogens but not for biotrophic pathogens [8]. That is, the future capability of a plant may be negatively affected when faced with an initial attack by insects, whereas initial infection by a biotrophic pathogen may positively influence subsequent attack by insects or necrotrophs. These instances highlight the importance of determining which plant-response variables are most suitable for assessing the priming benefit, such as the priming stimulus and its toxic concentration threshold. Therefore, plant priming is a multifaceted state that pre-empts defence responses against various environmental challenges, presenting a potential solution to enhance plant protection in agricultural systems [9]. Considering that there is a pressing need for new strategies that do not depend on fungicides or genetic engineering, the exploitation of the capacity of the plant immune system in association with other strategies may hold the potential to attain improved protection strategies for crops [10].

Strigolactones are natural compounds with more than 20 structural variants [11]. In the rhizosphere, strigolactones function as host detection cues to facilitate a mutualistic relationship between plants and arbuscular mycorrhizal (AM) fungi by stimulating pre-symbiotic branching [12]. This increases the probability of establishing a direct interaction between the fungus and the plant roots to encourage root colonization [13]. During this mycorrhizal interaction, nutrients such as phosphorus and nitrogen are exchanged by the fungus to stimulate plant growth through improved mineral nutrition, and in turn, the fungus obtains the necessary metabolites to bring its life cycle to fruition [14].

Combinations of strigolactones are exuded by some plant species, in varying concentrations, dependent on the plant developmental stage and condition, by both mycotrophic plants and non-mycotrophic plants, such as *Arabidopsis thaliana* and *Lupinus* species [15]. Such diversity in distribution leads to the hypothesis that strigolactones possess other functionalities, possibly affecting both beneficial and pathogenic soil microorganisms. Although it is generally accepted that plant hormones play positive roles in defence against necrotrophic pathogens, important exceptions to this classification exist [16]. As observed for some other plant hormones, strigolactones similarly exhibit different responses to different pathosystems. Previous studies have shown a range of reactions to the synthetic strigolactone GR24, varying from no change in radial growth and hyphal structure to an increase in hyphal branching with the suppression of radial growth [15,17]. Notably, these experiments were carried out using a combination of up to four stereoisomers of GR24, all with the potential to possess different biological activities [16]. The role of strigolactones in biotic stress responses may be associated with interactions with other phytohormones or hormone-dependent signalling. Belmondo et al. recently showed that a thioredoxin reductase is necessary for limiting *Botrytis cinerea* growth by GR24, indicating a potential relationship between strigolactones and reactive oxygen species (ROS) metabolism [18].

Initially, it was thought that host plants played a rather inactive role during the infection process of necrotrophic pathogens, but recently, it has been shown that the host plays a much larger role than initially anticipated [19]. Upon pathogen attack, cuticle penetration and primary lesion formation lead to an oxidative burst as an early defence response. This reaction begins with the rapid accumulation of ROS, which triggers a cascade of subsequent reactions, including the cross-linking of cell walls and the hypersensitive response (HR), resulting in the localized cell death at the sites of infection [20]. The oxidative burst functions as an effective response of plants against biotrophic pathogens, which rely on living host cells to bring their life cycle to fruition [21]. However, it does not protect the host against necrotrophs such as *B. cinerea*, in which plant cell death benefits the pathogen and leads to susceptibility. This hypothesis that necrotrophic fungi induce HR as part of their infection strategy has been supported by Dickman et al., who showed that transgenic tobacco lines expressing anti-apoptotic genes resulted in resistance to necrotrophic fungi [22].

The oxidative burst not only accumulates at the plant plasma membrane interface but also in the extracellular sheath of the fungal hypha [23]. *B. cinerea*, however, not only shows resistance against the oxidative burst but also produces ROS during the infection process, suggesting that ROS play a significant role in facilitating pathogen–host interactions [24]. Specifically, the appressoria of *B. cinerea* require the secretion of enzymes for successful penetration of the host tissue, amongst which superoxide dismutase (SOD) plays an important role in active cuticle penetration [25]. Bakshi et al. showed that SOD-deficient mutants exhibited reduced virulence on multiple hosts [26]. The source of superoxide acting as a substrate for BcSOD1 has yet to be determined. In an experiment conducted by Govrin and Levine it was shown that when leaves of *Arabidopsis thaliana* were infiltrated with a NADPH oxidase inhibitor before inoculation with *B. cinerea* spores, the resulting colonization of host tissue was significantly slower and was coupled with a decrease in ROS production [27]. The exact mechanism by which *B. cinerea* induces oxidative stress in its hosts is still unclear, but both fungal and host enzymes presumably contribute to the process [28]. Notably, the virulence of *B. cinerea* has been shown to correlate directly with the intensity of both the oxidative burst induced by the host as well as the accumulation of ROS by the fungus itself [20]. This occurrence is a highly regulated system which is dependent on the maintenance of redox homeostasis. The increase in ROS levels produced during the infection process affects the redox status of both the host and the fungus. The resulting balance between the production and scavenging of ROS needs to be maintained through ROS-detoxification systems to mitigate oxidative stress [24]. The ability of *B. cinerea* to effectively infect a wide host range primarily amounts to the capability of the fungus to counteract the broad range of plant defences through detoxification strategies by maintaining ROS homeostasis or through the secretion of antifungal compounds. Gaining a better understanding of the infection strategies of *B. cinerea* will aid in designing and implementing novel disease control strategies.

The aims of this study were to (i) assess the effects of the selected compounds on plant growth, (ii) evaluate the compounds for their potential ability to induce a priming response in tobacco plants against *B.cinerea,* (iii) determine whether these compounds are species specific by evaluating successful compounds against tissue culture grown tobacco and grapevine plants with the focus on establishing an accurate screening technique to evaluate the effects of priming at an early stage, particularly in slow growing plants such as grapevine, (iv) assess the direct effects of the strigolactone analogues (±)-GR24 and Nijmegen-1 on *B. cinerea* growth, and (v) evaluate the anti-oxidant profiles of *B. cinerea* in response to these compounds.

## 2. Results

### 2.1. Assessment of Non-Target Effects on Plant Growth following Chemical Treatment

**Figure 1:**
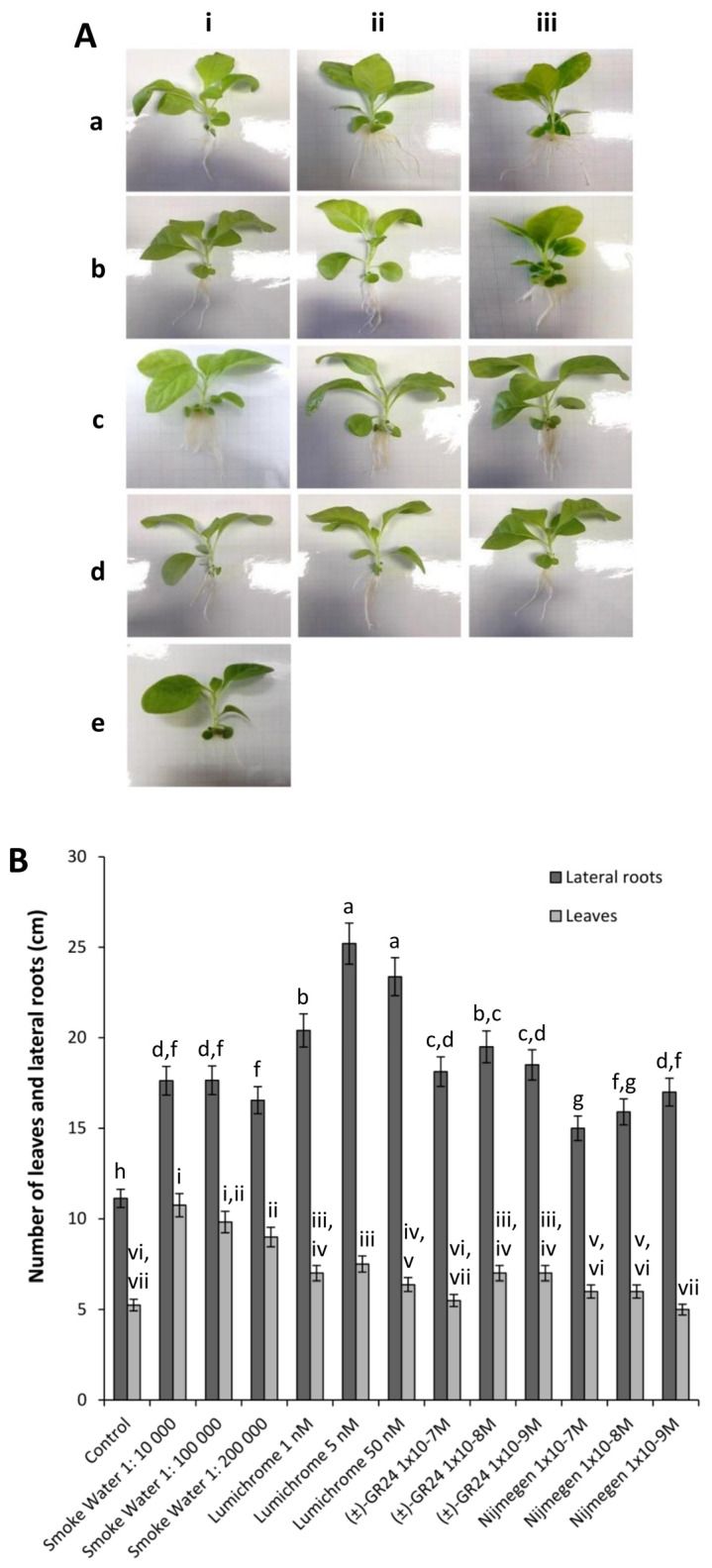
The effect of plant associated compounds on plant growth. (**A**) Plant growth and (**B**) lateral root and leaf development were measured in tobacco plants after 3 weeks of treatment under tissue culture conditions. Treatments were as follows: smoke water at concentrations of (i) 1:10,000, (ii) 1:100,000 and (iii) 1:200,000; lumichrome at concentrations of (i) 1 nM, (ii) 5 nM and (iii) 50 nM; (±)-GR24 at concentrations of (i) 1 × 10^−7^ M, (ii) 1 × 10^−8^ M and (iii) 1 × 10^−9^ M; Nijmegen-1 at concentrations of (i) 1 × 10^−7^ M, (ii) 1 × 10^−8^ M and (iii) 1 × 10^−9^ M; cultured in MS media (control). Error bars represent the means (±SE; n = 4) of two independent experiments. Letters a-h and numerals i-vii indicate significant differences.

Plant growth following chemical treatment was assessed for non-target effects. Germinated tobacco seedlings were grown in the presence of varying concentrations of smoke water, lumichrome, (±)-GR24 and Nijmegen-1. The effect of the exogenous application of compounds on lateral root formation and total leaf number was contrasted against the control without any compounds. In the presence of smoke water, the average lateral root formation increased by ca. 37%, while lumichrome increased lateral root formation by an average of ca. 45% compared to the control (Figure 1). Similar results were obtained for (±)-GR24 and Nijmegen-1, which were both able to increase lateral root formation by a cumulative average of ca. 40% and ca. 30%, respectively. No significant differences were observed in the number of leaves for lumichrome, (±)-GR24 and Nijmegen-1. Smoke water, however, increased the number of leaves formed by an average of ca. 47% (Figure 1). Interestingly, lumichrome was the only compound that resulted in stunted growth phenotypes at high concentrations, with plants taking on the appearance of proteoid roots with a clustered leaf formation between the first node and the stem (Figure 1). Phenotypic analysis showed that tobacco plants grown in the presence of smoke water, (±)-GR24 or Nijmegen-1 presented normal physiological development, across all concentrations, showing internodal symmetry and leaf surface areas equivalent or greater than the control plants. Root formation in the plants exposed to smoke water and Nijmegen-1 were similar, both resulting in thicker and longer main roots compared to the control plants (Figure 1). The (±)-GR24 treatment, however, induced the formation of long fibrous roots (Figure 1).

### 2.2. Fungal Inhibition Studies on Detached Leaves

**Figure 2:**
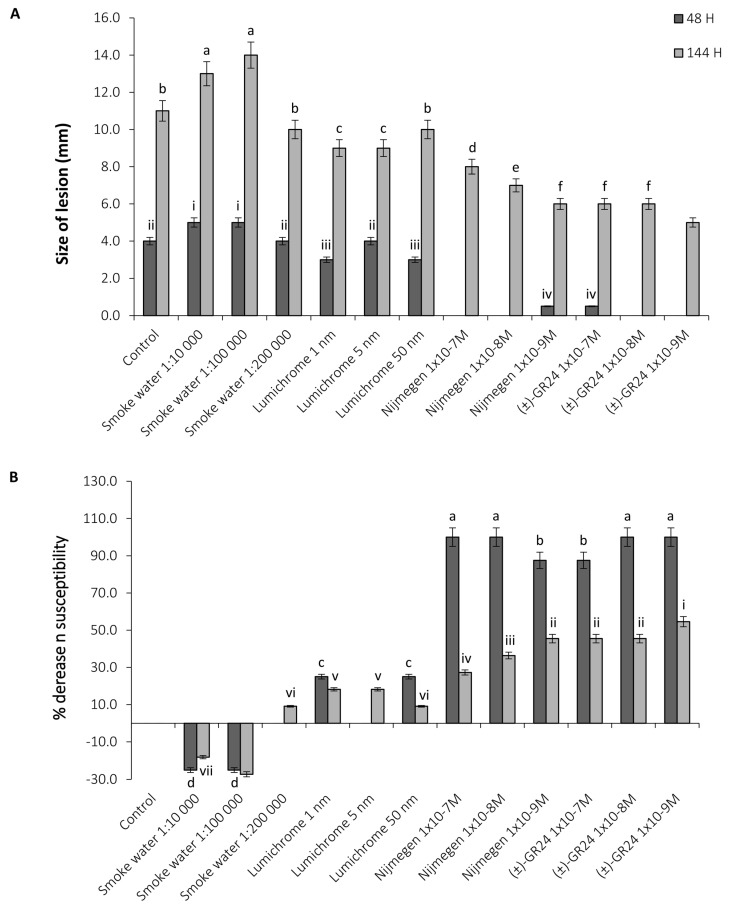
Disease susceptibility of treated tobacco plants to *B. cinerea*. A decrease in susceptibility to *B. cinerea* was determined by measuring the (**A**) size of lesions (mm) produced on detached leaves of the control and treated tobacco plants at 48 and 144 h post inoculation. (**B**) The susceptibility to *B. cinerea* was expressed as a percentage of the decrease in susceptibility normalised against the tolerance of the control set, which was referenced as 0%. Error bars represent the means (±SE; n = 4) of two independent experiments. Letters a-f and i-vii represent significant differences.

An adapted 10-point lesion index scale was used to define the range of lesion phenotypes in detached leaf priming screens (Figure S1) [29]. The smoke water and lumichrome-treated plants showed comparable disease progression patterns to the control throughout the experiment (Figures S2 and S3). Interestingly, the compounds Nijmegen-1 and (±)-GR24 both resulted in a 24 h delay in disease progression, with initial infection onset only occurring after 72 h (Figures S4 and S5). After 144 h, control tobacco leaves displayed lesions averaging 11 mm post infection with *B. cinerea* (Figure 2A). The final average lesion size between smoke water concentrations increased to an average size of 12 mm and increased to 10.3 mm in the lumichrome-treated plants. Nijmegen-1 and (±)-GR24, however, were able to reduce the infection lesions by ca. 40–50%, with final lesion sizes averaging 7 mm for Nijmegen-1 and 5 mm for (±)-GR24. Following characterization of disease lesions, the lesion sizes of all treated sets were standardized against the control to provide a percentage decrease in susceptibility (Figure 2B). Lumichrome was able to reduce susceptibility by an average of 20% at both time points, while smoke water was unable to decrease susceptibility at any of the concentrations tested. Nijmegen-1 and (±)-GR24 showed the highest percentage decrease in susceptibility, with a ca. 80–100% decrease 48 h post infection and a ca. 30–55% decrease 144 h post infection. Only Nijmegen-1 and (±)-GR24 were therefore selected for downstream analysis.

### 2.3. Anti-Oxidant Profiles of Infected Leaf Material

**Figure 3:**
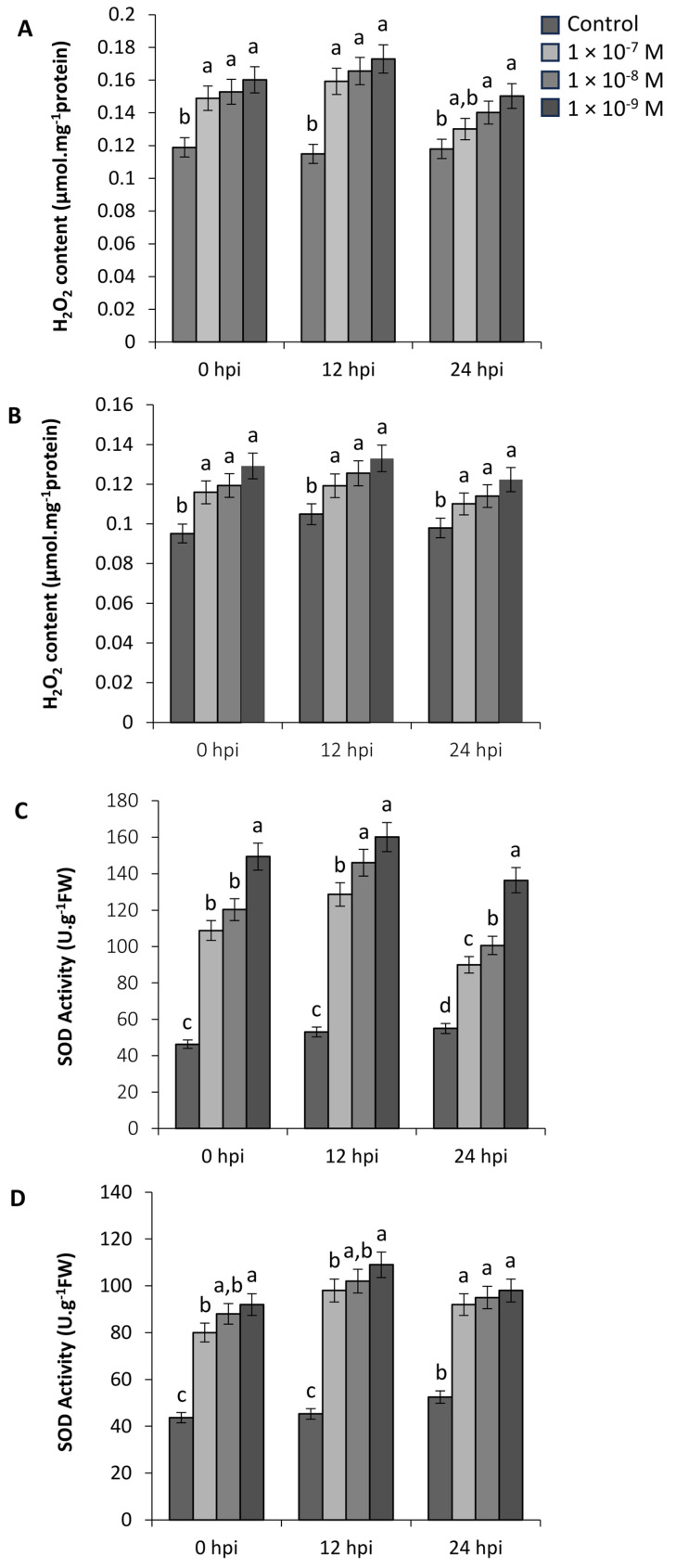
Effect of (±)-GR24 on superoxide content and hydrogen peroxide levels in tobacco leaves infected with *B. cinerea*. Measurements were performed on tobacco plants that were treated with (±)-GR24 for a period of 48 h. The H_2_O_2_ profiles for (±)-GR24 (**A**) and Nijmegen-1 (**B**) were determined via a spectrometric reading at 560 nm. The SOD profiles for (±)-GR24 (**C**) and Nijmegen-1 (**D**) were determined via a spectrometric reading at 550 nm. Values obtained were normalized with the fresh cell weights. Error bars represent the means (±SE; n = 4) of two independent experiments. Letters a-d represent significant differences.

The oxidative burst is among the early defence responses that are involved in inducing a primed response. The addition of the strigolactones (±)-GR24 and Nijmegen-1 as a root drench 48 h prior to infection triggered a single and transient burst of ROS in the detached leaves within a few hours of treatment (Figure 3). Levels of H_2_O_2_ were elevated for both strigolactone treatments at the initial point of 0 h post inoculation with *B. cinerea,* while SOD levels were ca. 50% higher compared to the control plants. The amount of H_2_O_2_ increased with the same kinetics for all concentrations for both (±)-GR24 and Nijmegen-1. A maximum concentration of H_2_O_2_ was reached 12 h post inoculation, reaching levels of 0.17 μmol.mg^−1^ of protein in response to a dose of (±)-GR24 at 1 × 10^−9^ M and at 0.13 μmol·mg^−1^ of protein in response to a dose of Nijmegen-1 at 1 × 10^−9^ M. Similar results were obtained for SOD determination, with both strigolactones exhibiting ca. 150% more SOD activity compared to the control 12 h post inoculation. The concentration response curves show that the oxidative burst occurred in a distinct stepwise manner until threshold. After this maximum, the relative amount of H_2_O_2_ and SOD declined to background levels 24 h post inoculation, and no further increases occurred until the end of experiment for either (±)-GR24 or Nijmegen-1.

### 2.4. In Vitro Plant Infection Screening

**Figure 4:**
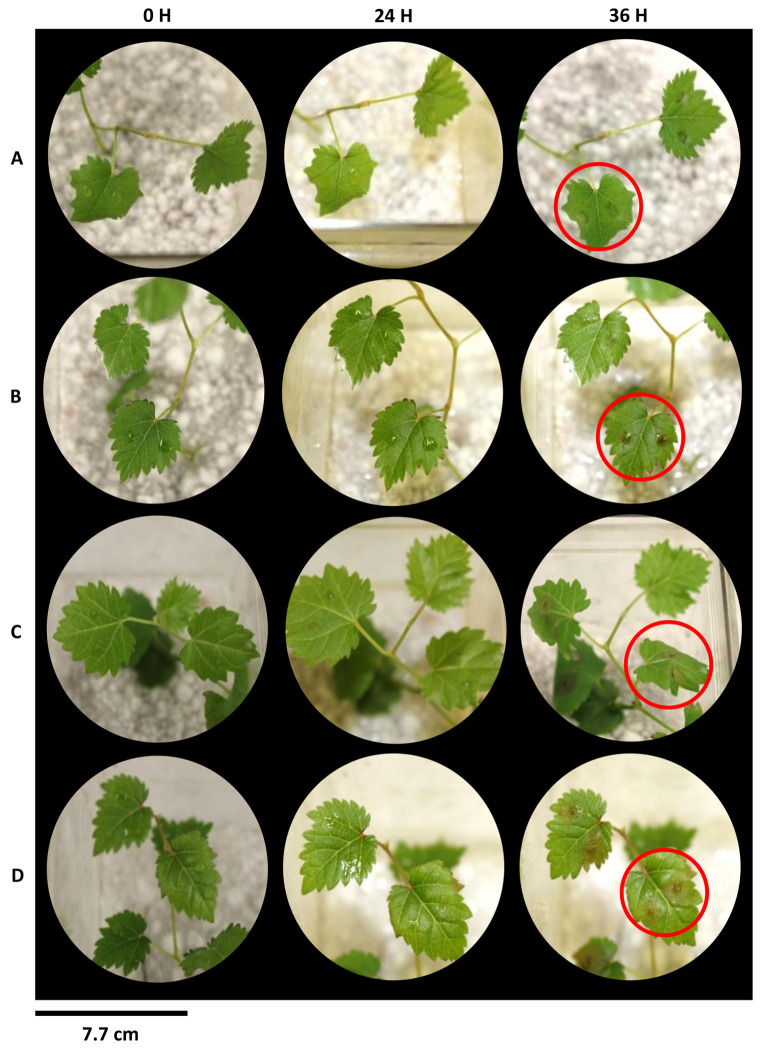
Disease symptom development of Nijmegen-1-treated tissue culture grapevine plants upon challenge with *B. cinerea*. Intact leaves were inoculated with a conidial suspension of *B. cinerea* and maintained at 22 °C at 100% humidity. Treatments consisted of root drench applications of water for (**A**) control sets or Nijmegen-1 at concentrations (**B**) 1 × 10^−7^ M, (**C**) 1 × 10^−8^ M and (**D**) 1 × 10^−9^ M. Images are representative of four biological repeats of two independent experiments. Disease development is indicated by the red circle.

**Figure 5:**
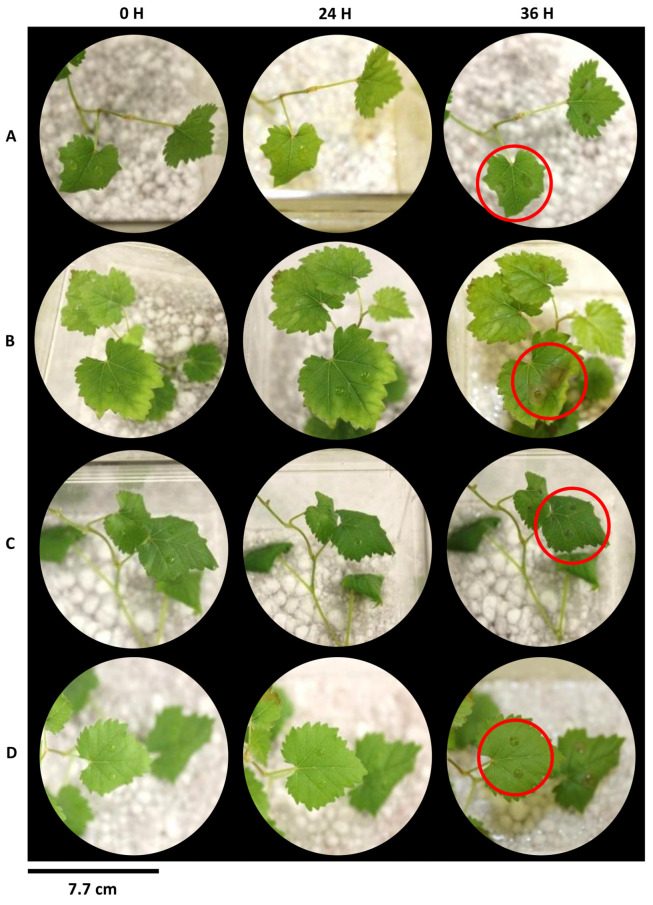
Disease symptom development of (±)-GR24-treated tissue culture grapevine plants upon challenge with *B. cinerea*. Intact leaves were inoculated with a conidial suspension of *B. cinerea* and maintained at 22 °C at 100% humidity. Treatments consisted of root drench applications of water for (**A**) control sets or (±)-GR24 at concentrations (**B**) 1 × 10^−7^ M, (**C**) 1 × 10^−8^ M and (**D**) 1 × 10^−9^ M. Images are representative of four biological repeats of two independent experiments. Disease development is indicated by the red circle.

The results obtained in the in vitro assay mirrored the pattern seen in the detached leaves of greenhouse grown tobacco plants (Figures S6 and S7). Leaves of control grapevine plants presented early signs of infection, including leaf wilting and actively growing hyphae. Overall, actively growing hyphae were not visible 36 h after inoculation across all concentrations for (±)-GR24 and Nijmegen-1 (Figure 4 and Figure 5).

### 2.5. Influence of (±)-GR24 and Nijmegen-1 on B. cinerea Radial Growth and Hyphal Branching

**Figure 6:**
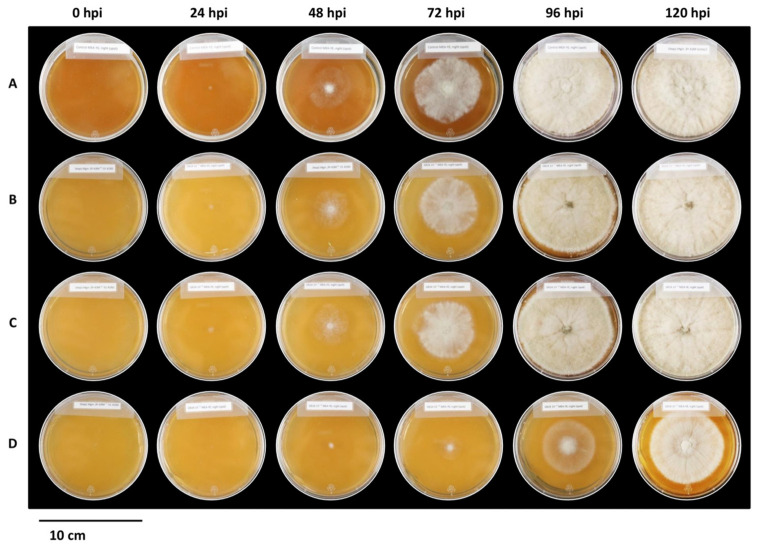
Effect of (±)-GR24 on the radial growth of *B. cinerea*. The radial growth of *B. cinerea* was assayed on MEA-YE agar (**A**) or in the presence of various concentrations of (±)-GR24 at (**B**) 1 × 10^−7^ M, (**C**) 1 × 10^−8^ M and (**D**) 1 × 10^−9^ M. Petri dishes were observed for 120 h post inoculation (hpi). Images are representatives of four replicates in each of two independent experiments.

The initial screening of *B. cinerea* against the concentrations of (±)-GR24 and Nijmegen-1 exhibited two disparate phenotypes for (±)-GR24 and Nijmegen-1, respectively. Almost no effect on radial growth of colonies was observed for any of the concentrations of Nijmegen-1 across all time points relative to the control (Figure S8) as well as the acetone solvent control. Inhibition of the fungal radial growth was, however, strongly evident at the (±)-GR24 concentration of 1 × 10^−9^ M at all the time points (Figure 6), while a weaker inhibition was also observed for (±)-GR24 concentrations of 1 × 10^−7^ M and 1 × 10^−8^ M. The fungal growth pattern was subsequently observed in detail under a stereomicroscope. Interestingly, one characteristic shared by the two strigolactones in the screening was a distinct effect on the hyphal morphology. Both strigolactone analogues resulted in impaired and abnormal branching structures compared to the hyphae of the control set (Figures S9 and S10). Notably, a quantitative dose–response relationship was exhibited for both strigolactones, with an increase in branching order being observed for the lower concentrations (Table 1). Branching in the control treatment was limited to the 2nd order, while exposure to (±)-GR24 triggered the formation of hyphal branches up to the 5th order (Figure S10). Nijmegen-1 yielded a similar pattern to (±)-GR24, but only induced hyphal branching up to the 4th order.

### 2.6. (±)-GR24 and Nijmegen-1 Affect the Formation of Infection Cushions

**Figure 7:**
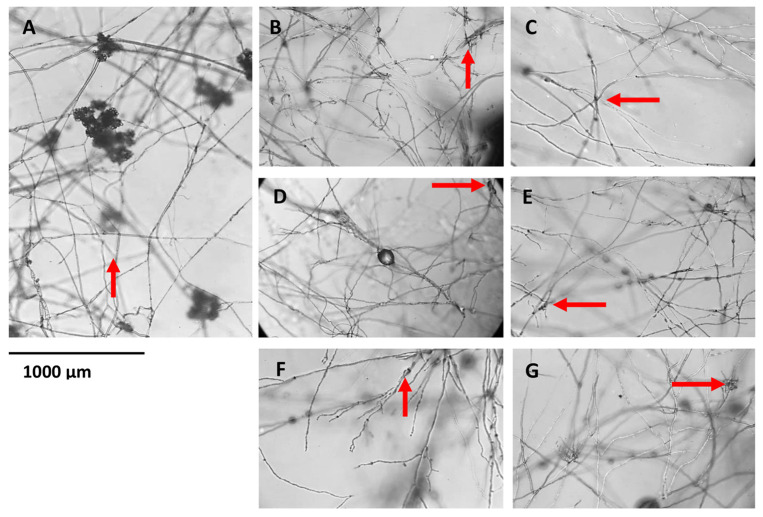
Effect (±)-GR24 and Nijmegen-1 on the infection cushion formation of *B. cinerea*. The growth of *B. cinerea* was assayed on 1% water agar (**A**) or in the presence of (±)-GR24 at concentrations of (**B**) 1 × 10^−7^ M, (**C**) 1 × 10^−8^ M and (**D**) 1 × 10^−9^ M and Nijmegen-1 at (**E**) 1 × 10^−7^ M, (**F**) 1 × 10^−8^ M and (**G**) 1 × 10^−9^ M. Petri dishes were observed under a stereomicroscope after 5 days post inoculation. Images are representatives of four replicates of two independent experiments. The formation of infection cushions is indicated with red arrows.

The ability of *B. cinerea* to form infection cushions, which function as penetration structures, in the presence of (±)-GR24 or Nijmegen-1 was evaluated via microscopic analysis of hyphae grown on solid media. The wild-type hyphae were able to form infection cushions (Figure 7), while the ability to form infection cushions was reduced in the hyphae grown in the presence of both (±)-GR24 and Nijmegen-1 across all concentration ranges of 1 × 10^−7^ M, 1 × 10^−8^ M and 1 × 10^−9^ M.

### 2.7. H_2_O_2_ and SOD Accumulation Is Altered by (±)-GR24 and Nijmegen-1

**Figure 8:**
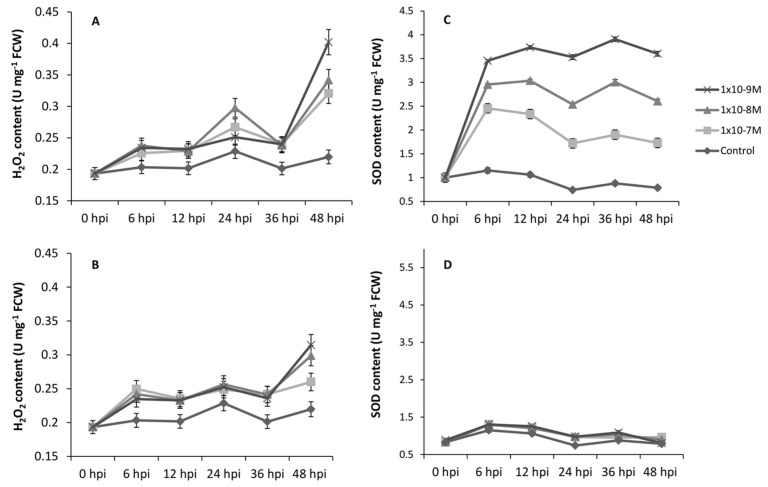
Effect of (±)-GR24 and Nijmegen-1 on the H_2_O_2_ and SOD profiles of *B. cinerea*. The H_2_O_2_ profiles for (±)-GR24 (**A**) and Nijmegen-1 (**B**) were determined via a spectrometric reading at 560 nm at intervals from 0–48 h post inoculation (hpi). The SOD profiles for (±)-GR24 (**C**) and Nijmegen-1 (**D**) were determined via a spectrometric reading at 550 nm. Values obtained were normalized with the fresh cell weights. Error bars represent the means (±SE; n = 4) of two independent experiments.

As a result of the decrease in infection cushion formation, we evaluated the potential link between the infection structures and ROS accumulation. The production of intracellular H_2_O_2_ and SOD levels were monitored in germinating hyphae grown in liquid medium. Upon exposure, little to no difference was observed in H_2_O_2_ levels amongst the concentration ranges from the initial point of inoculation up until 36 h post inoculation (hpi). However, between the time points of 36 and 48 h, a clear correlation between H_2_O_2_ content and strigolactone concentration became apparent for both (±)-GR24 and Nijmegen-1 (Figure 8). Notably, SOD accumulation was strongly induced across all concentrations and time points only for (±)-GR24, with a clear quantitative dose–response relationship between concentrations. Almost no fluctuations on intracellular SOD levels were observed for any of the concentrations of Nijmegen-1 across all time points relative to the control.

## 3. Discussion

This study investigated the potential priming effect of novel compounds against *B. cinerea* in *N. tabacum* and *V. vinifera* plants. Two of the four tested compounds, namely (±)-GR24 and Nijmegen-1, were rapidly perceived by both tobacco and grapevine plants and subsequently triggered inducible defence responses to produce a primed phenotype and were the focus of subsequent investigations. Nijmegen-1 and (±)-GR24 showed the highest percentage decreases in susceptibility with a ca. 80–100% decrease 48 h post infection and a ca. 30–55% decrease 144 h post infection. The application of these synthetic strigolactones also restricted lesion development by ca. 40–50% in the inoculated leaves within the initial hours of infection.

In this work, we have also shown the positive growth profiles of tobacco plants when exposed to varying concentrations of Nijmegen-1 and (±)-GR24. The concentrations examined are similar to those commonly used in in vitro germination experiments with root parasitic weeds such as *Striga* and *Orobanche* [30]. Higher strigolactone concentration ranges of 10^−6^–10^−7^ M have been previously tested and have also been shown to affect plant root branching, with similar results affecting root development [31]. There are currently little data available to support and quantify the actual amounts of strigolactones in the soil, but it is most likely that they are highly variable due to the rates of strigolactone secretion by plants, diffusion, adsorption and degradation [15]. It is most likely that plants and microorganisms are exposed to concentration gradients of strigolactones, resulting in differences in dose and composition profiles for each root system.

The synthetic strigolactones (±)-GR24 and Nijmengen-1 increased the induction of defence mechanisms, whereby the extent of these responses appeared to be associated with a single and transient burst of ROS. ROS accumulation has been used as one of the molecular markers in determining whether a plant is ready to offset a pathogen attack. Accordingly, pronounced ROS accumulation followed by pathogen challenge is one of the typical responses of primed plants. The generation of ROS is an essential instrument in pathogen recognition and functions as the initial point to restrict pathogen movement and reproduction. This effect was apparent in (±)-GR24- and Nijmegen-1-treated tobacco plants challenged with *B. cinerea*, in which disease symptoms were arrested for 24 h longer than the control plants. These results are in agreement with those findings observed by Aziz et al., whereby the addition of β-1,3-glucans to grapevine cell suspensions induced the production of H_2_O_2_, with a maximum level being reached 20 min after elicitation [32]. A study by Kauss et al. has also shown that pre-treatment of parsley suspension cell cultures with methyl jasmonate elicited the phenylpropanoid defence responses and primed the cultures for enhanced induction of the early oxidative burst [33].

These results suggest that ROS might play an important role in the ability of strigolactone analogues to trigger signals that can initiate defence pathways to boost plant immunity. The amounts of both SOD and H_2_O_2_ increased rapidly with the same step-wise progression between concentrations for both (±)-GR24 and Nijmegen-1. Although this burst was lower in the Nijmegen-1-treated set, when compared to (±)-GR24, there was significantly more SOD and H_2_O_2_ accumulation compared to the control plants. Interestingly, the activation of SOD and H_2_O_2_ was fortified and advanced in both (±)-GR24 and Nijmegen-1 compared to the control prior to the pathogen challenge. That is, SOD and H_2_O_2_ levels were activated during the 48 h priming period, after which a burst was observed 12 h post infection, which is consistent with the time frame at which direct fungal penetration occurs and the period at which most cell wall degrading enzymes are active [34]. It is possible that the strigolactones were able to trigger an initial ROS burst during the treatment phase to produce a standby defence response. That is, the subsequent basal levels were able to progress at concentrations strong enough to proactively inhibit early infection 12 h post infection with *B. cinerea*. These results suggest a primed state, in which a defence trigger has been sensitized rather than fully induced, allowing the plants to respond faster and more strongly following a challenge inoculation. Known priming agents, β-aminobutyric acid and sulfated laminarin, have been shown to induce resistance in grapevine against *Plasmopara viticola* through the priming of oxidative burst, callose deposition, phytoalexin synthesis and expression of the stilbene synthase [35].

Here, we could show that treating intact plants with synthetic strigolactone analogues resulted in local and systemic resistance against *B. cinerea* in detached leaves. The priming response observed in the detached leaves of both the (±)-GR24- and Nijmegen-1-treated tobacco plants indicates that these strigolactone analogues were capable of inducing a rapid and highly competent state, throughout the whole plant for a period of 48 h. In this study, we have also demonstrated the important influence of compound concentration on disease expression. Gaining a scientific understanding of hormone crosstalk is fundamental for designing effective protection strategies, as these form the regulatory steps that regulate a plant’s response to external stress. This is especially important as understanding crosstalk mechanisms and associated resistance trade-offs require knowledge of all the signals produced by the pathogen and the host that affect hormonal homeostasis. For example, Cipollini found that plants treated with high levels of salicylic acid exhibited significantly lower levels of seed production compared to those treated with lower concentrations, suggesting a tight correlation between compound dosage and fitness [36].

The effectiveness of (±)-GR24 and Nijmegen-1 as broad-spectrum priming agents was evaluated in vitro. Disease expression in vitro proved to be an effective proportional tool, which expressed comparable results to those observed under greenhouse conditions. That is, phenotypic disease development decreased compared with the control following treatment at all concentrations of both (±)-GR24 and Nijmegen-1 treatment. Similarly, the lowest concentration of 1 × 10^−9^ M for both strigolactone analogues proved to be the most effective in reducing symptom development, which were evaluated, based on the degree of wilting, browning and fungal hyphal occurrence. In vitro selection pressures experience minimal impact from the external environment and can precede and complement field trials. This approach may serve as a stand-alone screening tool to aid in further downstream analysis of slow-growing plants such as grapevine.

The effects of (±)-GR24 and Nijmegen-1 were also assessed on *B. cinerea*. Numerous reports exhibit conflicting data on the effect of the synthetic strigolactone GR24, on hyphal branching of a range of fungi, spanning from no effect to increased branching [15,37]. The synthetic strigolactones (±)-GR24 and Nijmegen-1 strongly induced hyper-branching in *B. cinerea*, with (±)-GR24 exerting a stronger effect by affecting colony diameter and radial growth. The main effects employed by the strigolactones included increased hyphal branching and elevated H_2_O_2_ and SOD levels, particularly for (±)-GR24. Besserer et al. reported that GR7 stimulates branching in the fungus *Gigaspora rosea* at concentrations as low as 10^−13^ M [12]. Likewise, Dor et al. saw similar results when GR24 was active at concentrations of 1 × 10^−5^ M and 1 × 10^−6^ M [15].

Similarly to other phytohormones, strigolactones are highly active at very low concentrations, which can be easily transported throughout the entire plant. Our experiments studied concentration ranges of 1 × 10^−7^–1 × 10^−9^ M with comparable results. These concentrations are also similar to those commonly used in in vitro germination experiments with root parasitic weeds, and therefore, the results observed at the lower concentrations could be accounted for as simulating the conditions within the natural environment. Our results are further supported by the findings of Rozpądek et al., who also observed inhibition of mycelial growth coupled with an accumulation of H_2_O_2_ following treatment with GR24 and similarly found the response of antioxidants was dependent on strigolactone concentration [38].

As SOD transforms one active oxygen species into another, specifically the conversion of O_2_ into H_2_O_2_, a concurrent induction of components of the anti-oxidative signalling pathway dealing with H_2_O_2_ is required to avoid oxidative damage. A low level of H_2_O_2_ produced by the NADPH oxidase (NOX) complex will partially regulate ROS and trigger fungal cell differentiation and development by regulating the redox-responsive pathways. Fungal strains with compromised ROS-detoxification systems have shown a severe reduction in virulence [24]. Therefore, coordinating signalling pathways is crucial for pathogenicity and the detoxification of cellular stress prompted by ROS. Our results suggest that strigolactones may activate the production of H_2_O_2_ either directly by inducing an oxidative burst, as with the increase in SOD with (±)-GR24, or indirectly by reducing the activity of ROS scavenging enzymes, as observed with the regulated levels of SOD following Nijmegen-1 treatment.

In plants, strigolactone signalling is mediated by the MAX2/SMAXL transduction pathway; however, no clear homologs of MAX2 have been identified in sequenced fungal genomes [38]. Therefore, the receptor-mediated mechanism for recognising strigolactones is unlikely to be the same receptor due to structural variations. It can be hypothesized that these unknown recognition patterns are key elements in determining whether the fungi are recognized as beneficial or pathogenic. These parallels in behaviour between racemic variants and potential differences in signalling pathways may account for the differences observed in the degree of effect on fungal hyphal branching between (±)-GR24 and Nijmegen-1.

Plants limit mycelium expansion within their tissues by triggering specific immune responses controlled by plant hormones. A relationship between strigolactones and these phytohormones has previously been suggested. Strigolactones have been shown to significantly upregulate the expression of genes encoding specific plant defensins [38]. Plant defensins have been reported to be involved in the induction of ROS to result in membrane permeabilization, which ceases the development of fungal cells. Calcium signalling has also been shown to be altered by defensins, which is necessary for normal fungal tip growth. Therefore, alterations in the signalling pathway can influence hyphal growth and induce hyper-branching of fungal hyphae [39].

The inability of *B. cinerea* to form infection cushions when exposed to the strigolactone analogues is comparable to results conducted by Sharman and Heale [40]. In brief, experiments were conducted in which *B. cinerea* cultures were grown under inductive stress conditions by removing nutrients from the inoculum. The results observed included impaired cushion formation and the inability to infect host cells. Furthermore, it has been previously illustrated that branching factors such as strigolactones may function in a way that dictates the formation of branch sites by accumulating key branching determinants in sub-apical hyphal cells until the threshold. Branching determinants that can induce the formation of new apical tips may include monomeric GTPases, calcium or ROS, but the exact mechanism by which strigolactones promote hyphal branching and the extent to which they act remains unclear [41]. Notably, reduced radial growth may be influenced by reduced apical dominance of the hyphal tip, leading to increased hyphal branching. An important characteristic associated with branching, specifically infection cushion formation, is virulence, in which strains that display stunted hyper-branching and irregular growth polarity exhibit reduced virulence in vivo [42].

Changes in the intracellular ROS levels are known to induce differentiation in *B. cinerea*. Specifically, the NOX complex, as a regulator of SOD, is necessary for appressoria development and fusion to form infection cushions. ROS function as signalling molecules at apposite concentrations but become detrimental to macromolecules and cellular processes when present in high concentrations [43]. The irregularities observed in cellular H_2_O_2_ and SOD levels may well account for the abnormalities observed in cellular development, particularly for infection cushion development. Overall, a potential direct mechanism that strigolactones may employ in fungi could include the increased accumulation of branching factors such as ROS to induce hyper-branching, resulting in the inability to form infection cushions coupled with decreased growth and virulence. At the same time, in plants, this is achieved by inducing the expression of genes which accumulate ROS as a by-product, such as defensins.

## 4. Materials and Methods

### 4.1. Plant Growth Conditions

Wild-type *Nicotiana tabacum* SR1 cv Petit Havana (WT tobacco) was used as the host plant for experimental investigation. Seeds were surface sterilized according to the vapour-phase sterilization method of Clough and Bent [44]. For germination, seeds were dispersed over 100 mm Petri dishes containing MS media [45] supplemented with 15 g/L sucrose and solidified using 1% agar, and left in the dark for 3 d to promote synchronous germination. After 3 d, seeds were transferred to the light and incubated at 26 ± 2 °C with a 16 h light/8 h dark photoperiod under a light intensity of 50 μmol photons.m^−2^.s^−1^ (400–700 nm wavelength) using two Osram L58W/640 energy saver lamps for 3 weeks. For priming studies, in vitro-grown tobacco seedlings were hardened off in a greenhouse growth room for 2 weeks under a 25/19 ± 2 °C day/night temperature cycle under a 16 h light/8 h dark cycle, at a photosynthetic photon flux density of 50 μmol photons.m^−2^.s^−1^ (400–700 nm wavelength) using two Osram L58W/640 energy saver lamps. Plants were supplemented with nutrient solution (1 mM K_2_SO_4_; 2 mM Mg SO_4_; 5 mM CaCl_2_; 5 mM KNO_3_; 10 mM NH_4_NO_3_; 1 mM K_2_HPO_4_ buffer at pH 6.4; 5 μM H_3_BO_3_; 5 μM MnSO_4_; 1 μM ZnSO_4_; 1 μM CuSO_4_; 2 μM Na_2_MoO_4_; 1 μM CoSO_4_; 100 μM Fe-NaEDTA and 10 mM 4-(2-hydroxyethyl)-1-piperazineethanesulfonic acid (HEPES) at pH 6.4) at the V1 stage (two fully expanded leaves and one emerging leaf). Plants at the V2 stage of development (four fully expanded leaves and one emerging leaf) with similar heights were selected for all subsequent priming experiments.

### 4.2. Chemical Treatment for Plant Growth Studies

To evaluate the growth effects of the target compounds, tobacco seedlings were germinated as described above and maintained on a basal medium of solid MS supplemented with the following: 1:10,000, 1: 100,000 and 1:200,000 smoke water; 1 nM, 5 nM and 50 nM lumichrome; 1 × 10^−7^ M, 1 × 10^−8^ M and 1 × 10^−9^ M (±)-GR24; 1 × 10^−7^ M, 1 × 10^−8^ M and 1 × 10^−9^ M Nijmegen-1, respectively. The tobacco plants were incubated at 26 ± 2 °C with a 16 h light/8 h dark photoperiod at a photosynthetic photon flux density of 50 μmol photons.m^−2^.s^−1^ (400–700 nm wavelength) using two Osram L58W/640 energy saver lamps for 3 weeks. After 3 weeks, the number of lateral roots and leaves was evaluated and recorded against the control plants.

### 4.3. B. cinerea BO5.10 Growth and Cultivation

*B. cinerea* strain BO5.10 was the pathogenic agent used for experimental investigation due to its recently published genome sequence, which facilitates the ease of application of molecular techniques. To prepare the *B. cinerea* spore suspension, 1 μL of spore stock was inoculated onto MEA (Malt Extract Agar) supplemented with 1% (*w*/*v*) yeast extract. Plates were incubated in the dark at 25 ± 2 °C until mycelia had reached the edge of the plates. Plates were then exposed to UV-light for 2 d at a photosynthetic photon flux density of 50 μmol photons.m^−2^.s^−1^ (400–700 nm wavelength) using two Osram L58W/640 energy saver lamps at 25 ± 2 °C, after which they were returned to the dark and left for 2 weeks at 25 ± 2 °C before spores were harvested. Spores were harvested by adding sterile water to each plate, and gently scraping the surface with a sterile loop to release the spores. Using distilled water, the spores were filtered through glass wool [46]. The spore suspension was allowed to hydrate for 16 h at 4 °C. Germination potential was determined microscopically following incubation of the spore suspension onto 1% (*w*/*v*) water agar for 16 h at room temperature. The spore concentration was determined with a counting chamber (Marienfeld, Germany) and adjusted to a final concentration of 10^6^ spores/mL using 50% filter-sterilized grape juice.

### 4.4. Chemical Treatment and Plant Inoculation for Priming Studies

For priming studies, whole tobacco plants were transferred to infection chambers and treated with the respective compound 48 h prior to infection to facilitate acclimation to 100% humidity at 22 ± 2 °C. Target compounds were prepared to specification and applied as 20 mL soil drench applications at three different concentrations and control plants treated with distilled water. To investigate the presence of priming, inoculation with *B. cinerea* was conducted on detached leaves of the treated plants, using the two youngest fully expanded leaves for inoculation. Detached leaves were placed with the adaxial surface in contact with dampened Whatman paper discs in Petri dishes, and the spore suspension was deposited on fixed positions left and right of the midvein. Disease progression was evaluated through macroscopic observation of the diameter of necrosis over a 6 d period and results expressed as necrosis size in square millimetres (mm^2^). Only those compounds exhibiting distinct priming phenotypes were selected for downstream analyses, during which infection assays were repeated in duplicate and the infected leaf material harvested, pooled, and flash frozen at time points 0, 12 and 24 h post infection.

### 4.5. Measurement of Hydrogen Peroxide (H_2_O_2_) Activity

H_2_O_2_ content was determined based on a previously described method [47]. For determination of H_2_O_2_ content, 0.5 g of frozen leaf material was homogenized in 1 mL of solution containing 10% trichloroacetic acid (TCA), 5 mM K_2_HPO_4_ pH 5.0 and 0.5 M KI. The suspensions were centrifuged at 12,000× *g* for 15 min at 4 °C, and 200 μL of the supernatant was retained for analysis. Samples were incubated at 25 ± 2 °C for 20 min prior to analysis of the absorbance readings taken at 390 nm. H_2_O_2_ content was calculated based on a standard curve constructed from the absorbance (A390 nm) of H_2_O_2_ standards prepared in 0.1% TCA.

### 4.6. Measurement of Superoxide Dismutase (SOD) Activity

Superoxide dismutase activity was assayed in accordance with the nitroblue tetrazolium (NBT) method of Zhou et al. [48]. The reaction mixture contained 0.5 g of leaf tissue homogenized in 5 mL extraction buffer consisting of 50 mM phosphate (pH 7.8), 26 mM methionine, 750 μM NBT, 1 μM EDTA and 20 μM riboflavin. The photoreduction of NBT was determined at 560 nm, where one unit of SOD was defined as 1 μmol of enzyme that reduces NBT to purple formazan by 50%. SOD activity was expressed as enzyme units per gram fresh weight (U g^−1^ FW).

### 4.7. Tissue Culture Growth and Infection Assays

Grapevine explants containing a single node with axillary buds, derived from a mother plant (cv. Cabernet Sauvignon), were surface sterilized. In brief, each explant was washed twice with autoclaved water and then immersed in 70% ethanol. The material was transferred to a sterilisation solution consisting of 0.5% (*w*/*v*) sodium hypochlorite with 1–2 drops of Tween-20. The explants were maintained on a basal medium of perlite containing liquid MS media supplemented with, 30 g/L sucrose at pH 5.8, and incubated at 26 ± 2 °C with a 16 h light/8 h dark photoperiod under a light intensity of 50 μmol photons.m^−2^.s^−1^ (400–700 nm wavelength) using two Osram L58W/640 energy saver lamps for 4 weeks. Vapour-phase decontaminated tobacco seedlings were established under the same basal growth conditions. For infection studies, completely hardened, fast-growing grapevine and tobacco plants were transferred to fresh growth medium, consisting of equal ratios of sterile vermiculite and perlite. To facilitate ex vitro acclimatization, magenta lids were used to gradually reduce the relative humidity to ambient levels by tilting the lids further open every second day for 1 week. Plants were treated with the respective compound 48 h prior to infection as 20 mL root drench applications at three different concentrations and control plants treated with distilled water. Tissue culture plants underwent the same infection protocol as previously described.

### 4.8. B. cinerea Branching Bioassay and Growth Response 

*B. cinerea* spores were inoculated into 50 mL ME (30 g/L malt extract and 5 g/L peptone) in 150 mL Erlenmeyer flasks to a final concentration of 106 spores/mL. The cultures were maintained on a rotary shaker (200 rpm) at 25 °C. To evaluate the potential direct effects of (±)-GR24 and Nijmegen-1 on *B. cinerea* growth, an in vitro assay was performed. Stock solutions (1 × 10^−7^ M) of racemic (±)-GR24 and Nijmegen-1 were prepared by initially dissolving the specific molecule in 100 μL acetone and then diluting to a concentration of 1 × 10^−8^ M and 1 × 10^−9^ M with sterile ddH2O. Stock solutions were made fresh prior to all assays. For growth and branching assays, 1 μL of spore suspension was applied to the centre of both MEA and 1% water agar plates, each supplemented with varying (±)-GR24 and Nijmegen-1 concentrations. Three different concentrations of (±)-GR24 and Nijmegen-1 were analysed, namely 1 × 10^−7^ M, 1 × 10^−8^ M and 1 × 10^−9^ M. Plates were incubated in the dark at 25 °C until mycelia had reached the edge of the plates. Plates were then exposed to UV-light for 48 h at a photosynthetic photon flux density of 50 μmol photons.m^−2^.s^−1^ (400–700 nm wavelength) using two Osram L58W/640 energy saver lamps at 25 ± 2 °C, after which they were returned to the dark. For the analysis of fungal growth and development, images were taken of the MEA fungal plates in 24 h intervals from the start of inoculation for a 5 d period. For analysis of branching and infection cushion formation, the 1% water agar fungal plates were examined under a stereomicroscope, and images of the infection cushions and fungal branching structures were captured. The number of hyphal branches of the second, third, fourth and fifth orders, as well as total number of branches, was recorded for a 1000 μm length of each primary branch beginning at the youngest hyphal tip. The experiments were conducted twice with four biological replicates in each experiment. Presented data are representative from one typical experiment. All experiments were run in parallel using controls systematically prepared with the corresponding amount of acetone solvent.

### 4.9. Anti-Oxidant Detection Assays 

Determination of anti-oxidant activity was achieved in quantitative liquid assays. Pre-cultures were inoculated in triplicate into 50 mL malt extract containing the three different concentrations of (±)-GR24 and Nijmegen-1 in 125 mL Erlenmeyer flasks. Cultures were harvested after 24 h via centrifugation at 17,000× *g* for 3 min, and the pellet was weighed to determine the fresh weight of the mycelium. The pellet was re-suspended in 50 mM phosphate buffer pH 7.0 and homogenized using glass beads. The suspension was re-centrifuged for 10 min at 17,000× *g*, and the supernatant used in the subsequent anti-oxidant assays as a source of intracellular enzyme.

#### 4.9.1. Measurement of Superoxide Dismutase (SOD) Activity 

The determination of SOD activity was based on the previously described method of Spychalla and Desborough [49]. The reaction mixture contained 50 mM Na_2_CO_3_ (pH 10.2), 0.1 mM EDTA, 0.01 mM ferricytochrome c, 0.05 mM xanthine and 100 μL of the intracellular enzyme fraction. The rate of ferricytochrome c reduction was determined based on the absorbance at 550 nm. Enzyme activities were expressed as units per milligram fresh cell weight (U/mg FCW) where one unit was defined as 1 μmol of enzyme which inhibited the rate of ferricytochrome c reduction by 50%.

#### 4.9.2. Measurement of Hydrogen Peroxide (H_2_O_2_) Activity 

The determination of H_2_O_2_ activity on the previously described method of Chen et al., with slight modifications [50]. A 500 μL aliquot of the intracellular enzyme fraction was added to the reagent mixture consisting of 500 μM ferrous ammonium sulfate, 50 mM H_2_SO_4_, 200 μM xylenol orange, and 200 mM sorbitol and incubated at room temperature for 30 min. The peroxide-mediated oxidation of ferrous to ferric was determined by measuring the absorbance of the ferric-xylenol orange complex at 560 nm. Enzyme activities were expressed as units per milligram fresh cell weight (U/mg FCW) where one unit was defined as 1 μmol of enzyme that catalyses the formation of 1 mM H_2_O_2_/h.

### 4.10. Statistical Analysis

All experiments were performed independently two times, each with four biological representatives per data set. Data were presented as mean ± standard error of four independent determinations. For statistical analysis, one-way analysis of variance (ANOVA) test was used for all data.

## 5. Conclusions

In conclusion, these results show the potential of strigolactones as functional plant priming agents for accelerated ROS production, leading to increased resistance to *B. cinerea*. Heightened disease perturbation without a causal agent confirms the alternative role of (±)-GR24 and Nijmegen-1 as plant defence activators. These results strongly suggest that ROS is a mechanism used for priming by both (±)-GR24 and Nijmegen-1. In addition to their priming activity, strigolactones are active inducers of hyper-branching in roots, making them an appealing alternative to chemical fungicides [51]. We demonstrated that (±)-GR24 and Nijmegen-1 can influence ROS levels, providing further confirmation that ROS are likely intermediaries of strigolactone actions. These results demonstrated that ROS homeostasis and regulation are imperative to support regular development and potentially establish the virulence of *B. cinerea*. This study has further shown the complexity and the vast number of potential mechanisms employed by strigolactones. Future work in the form of comprehensive enzyme and anti-oxidant activity assays, using the purified enantiomers of GR24, could reveal the apparent effect of GR24 on defining key determinants of the growth and virulence of *B. cinerea*, in addition to elucidating the potential mode of actions of strigolactones in this regard. These findings support the hypothesis that subsequent pathogen challenges triggered augmented molecular and cellular defence-related responses in strigolactone-treated plants. Along with conventional bio-control agents, strigolactones could provide novel disease control strategies that satisfy environmental regulations.

## Figures and Tables

**Table 1:** Hyphal branching patterns of *B. cinerea* following exposure to (±)-GR24 and Nijmegen-1.

			(±)-GR24			Nijmegen-1		
Order of Branching	Water Control	Solvent Control	1 × 10^−7^ M	1 × 10^−8^ M	1 × 10^−9^ M	1 × 10^−7^ M	1 × 10^−8^ M	1 × 10^−9^ M
2nd	3	2	5	9	12	7	15	17
3rd	1	1	3	4	8	3	3	7
4th	0	0	3	4	7	2	2	3
5th	0	0	0	1	3	0	0	0

Data presented in this table are the means ± standard error of three replicates (n = 3). The total number of hyphal branches was recorded on a total length of 1000 µm, starting from the end of the youngest hyphal tip.

## Data Availability

Data are available upon request from the corresponding author.

## Supplementary Materials

The following supporting information can be downloaded at: https://www.mdpi.com/article/10.3390/plants12183202/s1, Figure S1: Lesion index scale; Figure S2: Disease symptom development in detached leaves of smoke water-treated tobacco plants upon challenge with *B. cinerea*; Figure S3: Disease symptom development in detached leaves of lumichrome-treated tobacco plants upon challenge with *B. cinerea*; Figure S4: Disease symptom development in detached leaves of Nijmegen-1-treated tobacco plants upon challenge with *B. cinerea*; Figure S5: Disease symptom development in detached leaves of (±)-GR24-treated tobacco plants upon challenge with *B. cinerea*; Figure S6: Disease symptom development of Nijmegen-1-treated tissue culture tobacco plants upon challenge with *B. cinerea*; Figure S7: Disease symptom development of (±)-GR24-treated tissue culture tobacco plants upon challenge with *B. cinerea*; Figure S8: Effect of Nijmegen-1 on the radial growth of *B. cinerea*; Figure S9: Effect of Nijmegen-1 on the hyphal branching structures of *B. cinerea*; Figure S10: Effect of (±)-GR24 on the hyphal branching structures of *B. cinerea*.
