# Supplementary material for: Strigolactones GR-24 and Nijmegen Applications Result in Reduced Susceptibility of Tobacco and Grapevine Plantlets to Botrytis cinerea Infection
Source: Plants (Basel). 2023 Sep 7;12(18):3202. doi: 10.3390/plants12183202 (PMC10535315; doi:10.3390/plants12183202)
Supplement: Supplementary file 1 [file plants-12-03202-s001.zip › MDPI Plants (2023) Strigolactone Vogel_supplementary_2023-07-07 JM_PH edits JM.pdf]

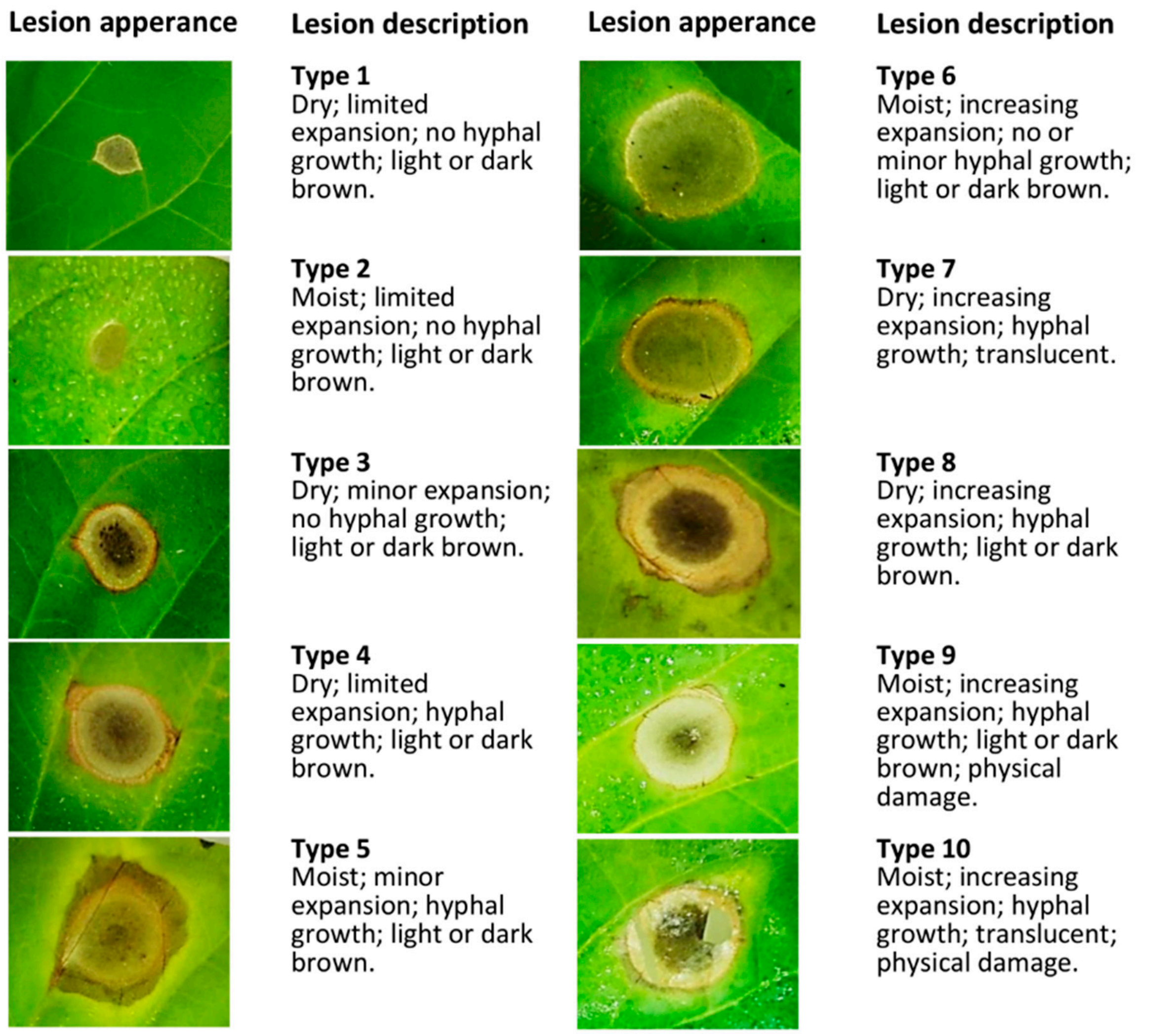

Figure S1. Lesion index scale and lesion descriptions used to define tolerance and susceptibility after infection of tobacco with B. cinerea. Type 1 lesions indicate the most tolerant phenotype, whereas type 10 represents the most susceptible phenotype. Adapted from Carstens et al., (2003)[1].

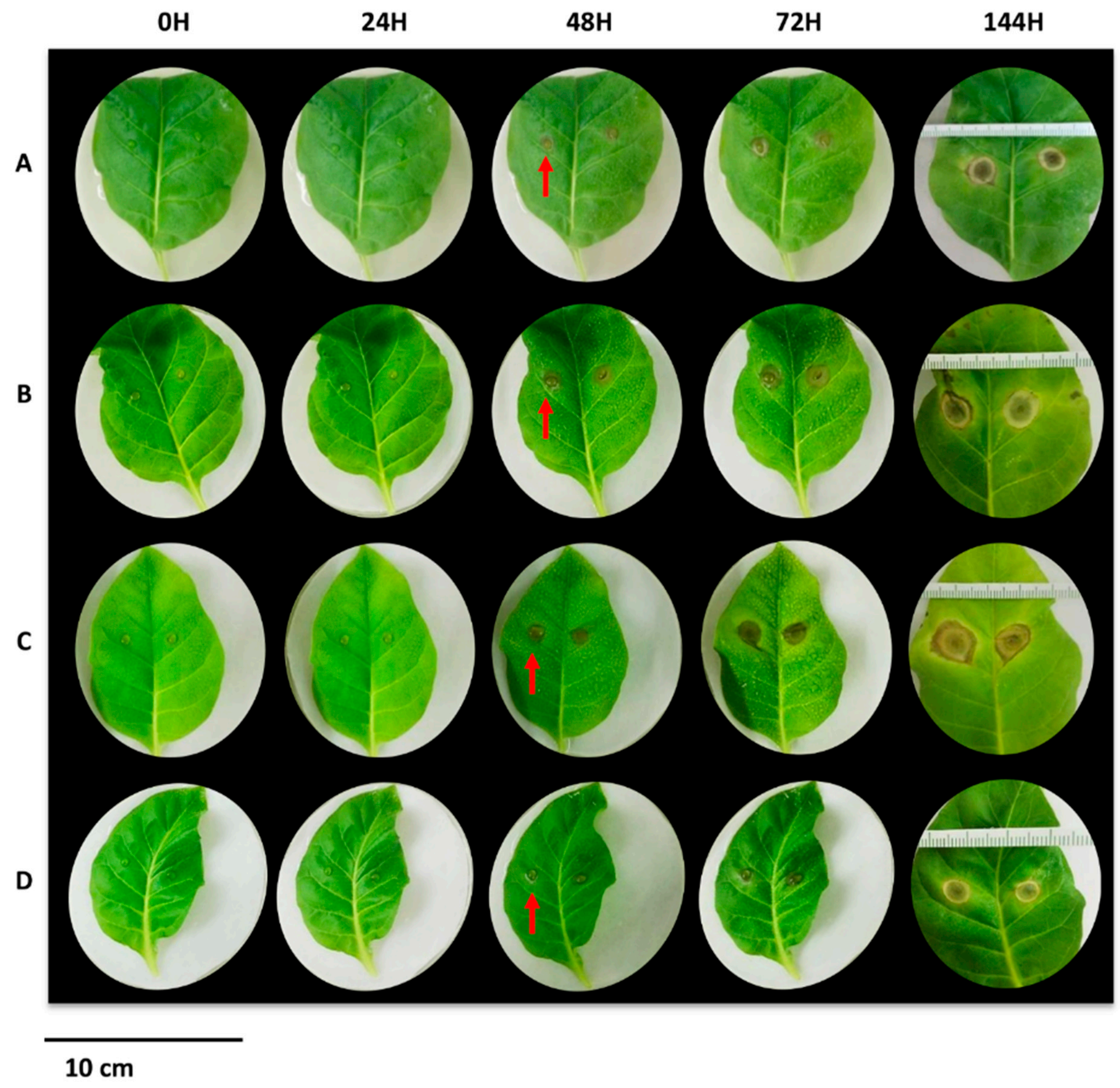

Figure S2. Disease symptom development in detached leaves of smoke water treated tobacco plants upon challenge with *B. cinerea*. Excised leaves were inoculated with a conidial suspension *B. cinerea* conidial suspension and maintained at 22°C at 100% humidity. Treatments consisted of root drench applications of water for (A) control sets or smoke water at concentrations (B) 1:10 000, (C) 1:100 000 and (D) 1: 200 000. Necrotic lesions were photographed and categorized according to a 10-point lesion scale 6 days post inoculation. Images are representative of four biological repeats of two independent experiments. Initial disease development is indicated by the red arrow.

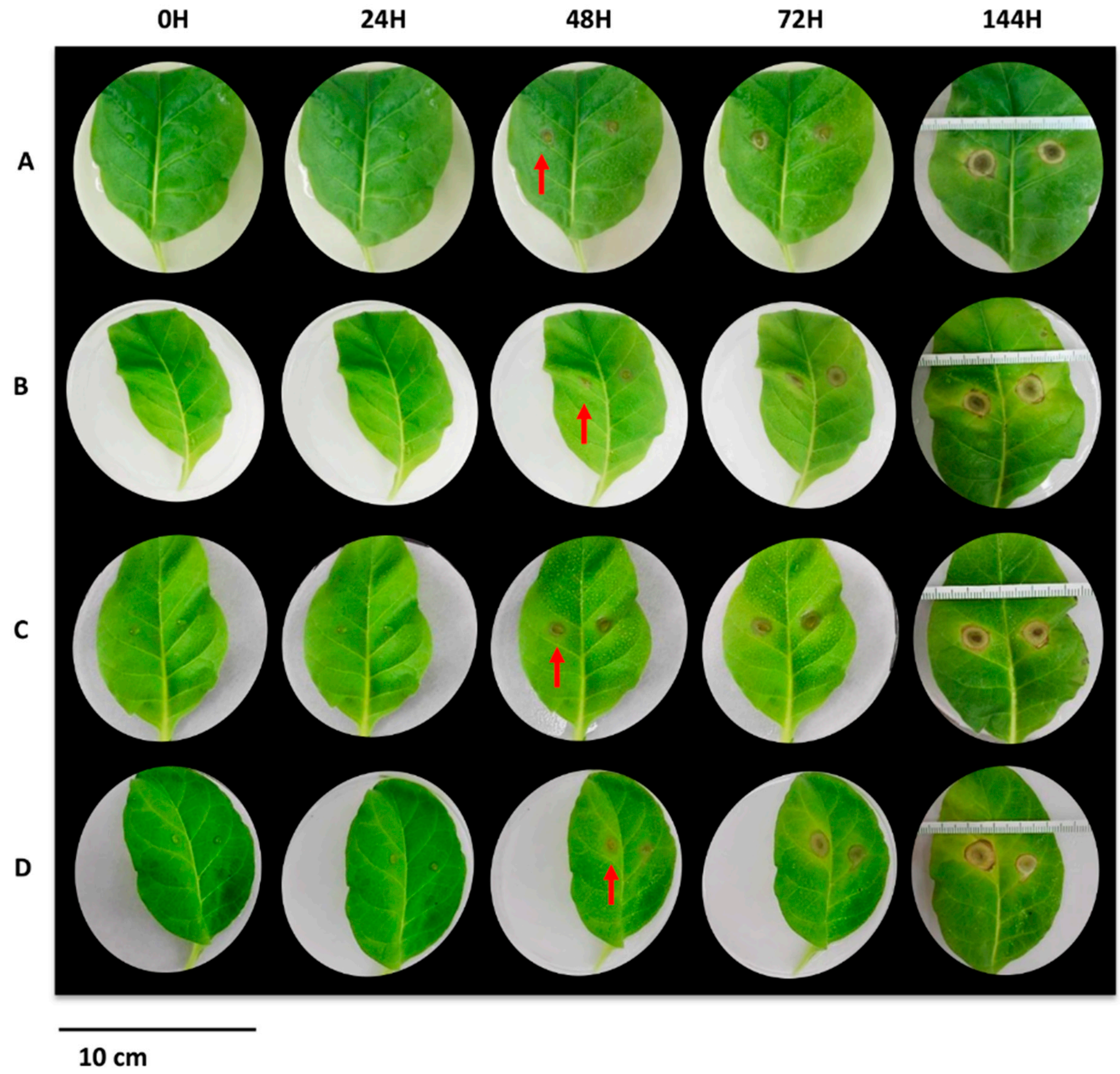

Figure S3. Disease symptom development in detached leaves of lumichrome treated tobacco plants upon challenge with *B. cinerea*. Excised leaves were inoculated with a conidial suspension of *B. cinerea* and maintained at 22°C at 100% humidity. Treatments consisted of root drench applications of water for (A) control sets or lumichrome at concentrations (B) 1nM, (C) 5nM and (D) 50nM. Necrotic lesions were photographed and categorized according to a 10-point lesion scale 6 days post inoculation. Images are representative of four biological repeats of two independent experiments. Initial disease development is indicated by the red arrow.

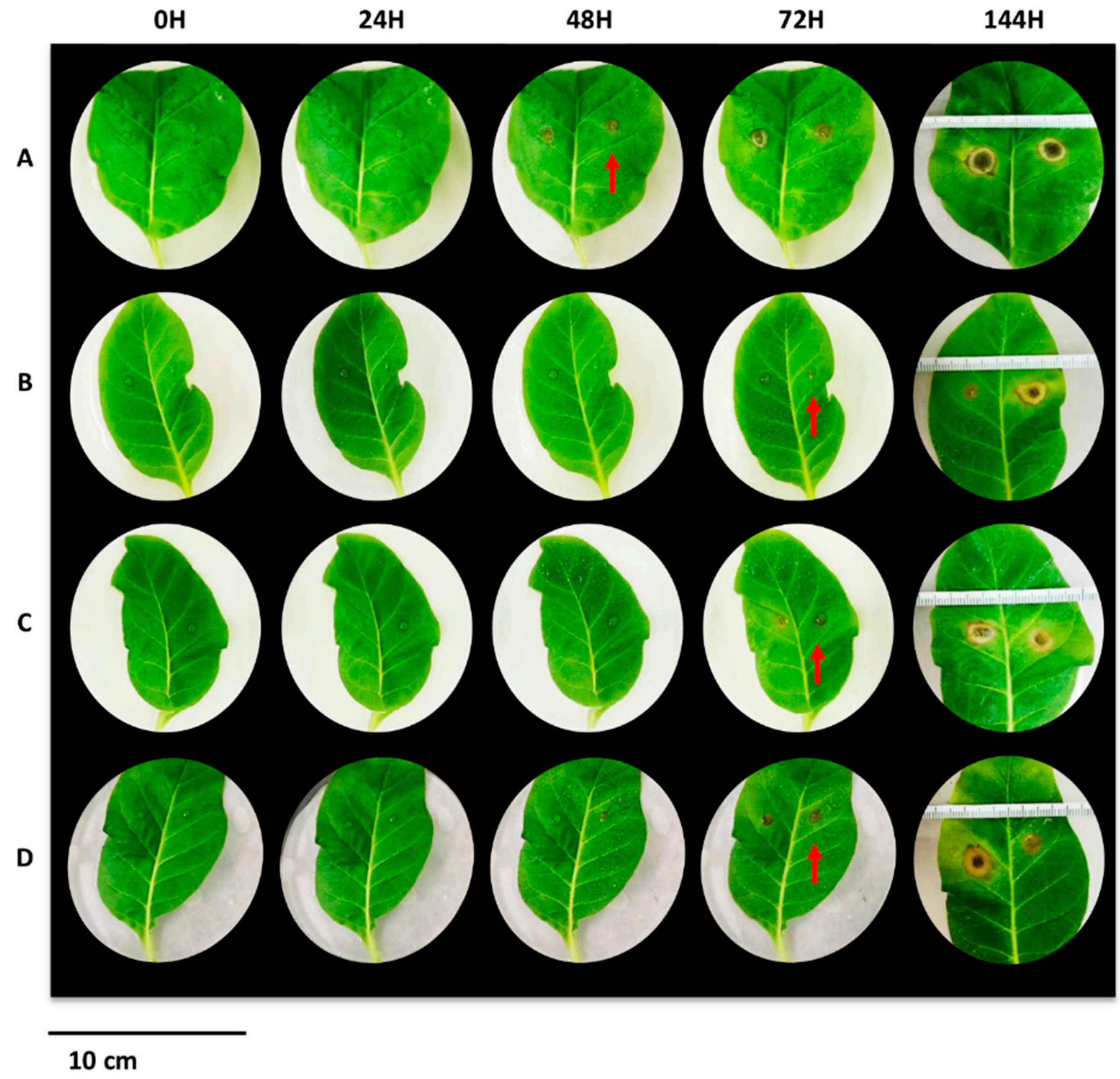

Figure S4. Disease symptom development in detached leaves of Nijmegen-1 treated tobacco plants upon challenge with *B. cinerea*. Excised leaves were inoculated with a conidial suspension of *B. cinerea* and maintained at 22°C at 100% humidity. Treatments consisted of root drench applications of water for (A) control sets or Nijmegen-1 at concentrations of (B) $1x10^{-7}$M, (C) $1x10^{-8}$M and (D) $1x10^{-9}$M. Necrotic lesions were photographed and categorized according to a 10-point lesion scale 6 days post inoculation. Images are representative of four biological repeats of two independent experiments. Initial disease development is indicated by the red arrow.

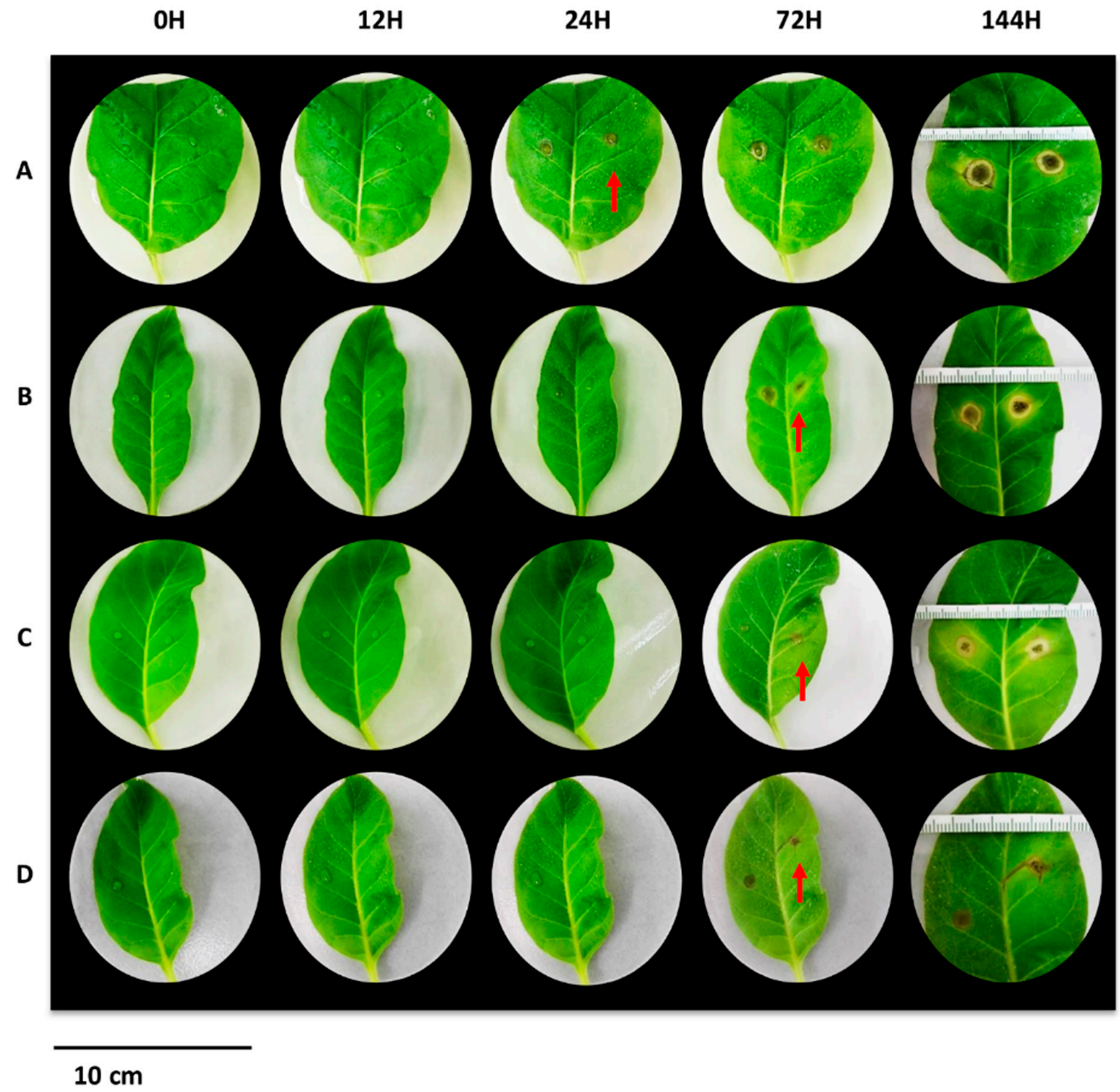

Figure S5. Disease symptom development in detached leaves of (±)-GR24 treated tobacco plants upon challenge with *B. cinerea*. Excised leaves were inoculated with conidial suspension of *B. cinerea* and maintained at 22°C at 100% humidity. Treatments consisted of root drench applications of water for (A) control sets or (±)-GR24 at concentrations of (B) $1x10^{-7}$M, (C) $1x10^{-8}$M and (D) $1x10^{-9}$M. Necrotic lesions were photographed and categorized according to a 10-point lesion scale 6 days post inoculation. Images are representative of four biological repeats of two independent experiments. Initial disease development is indicated by the red arrow.

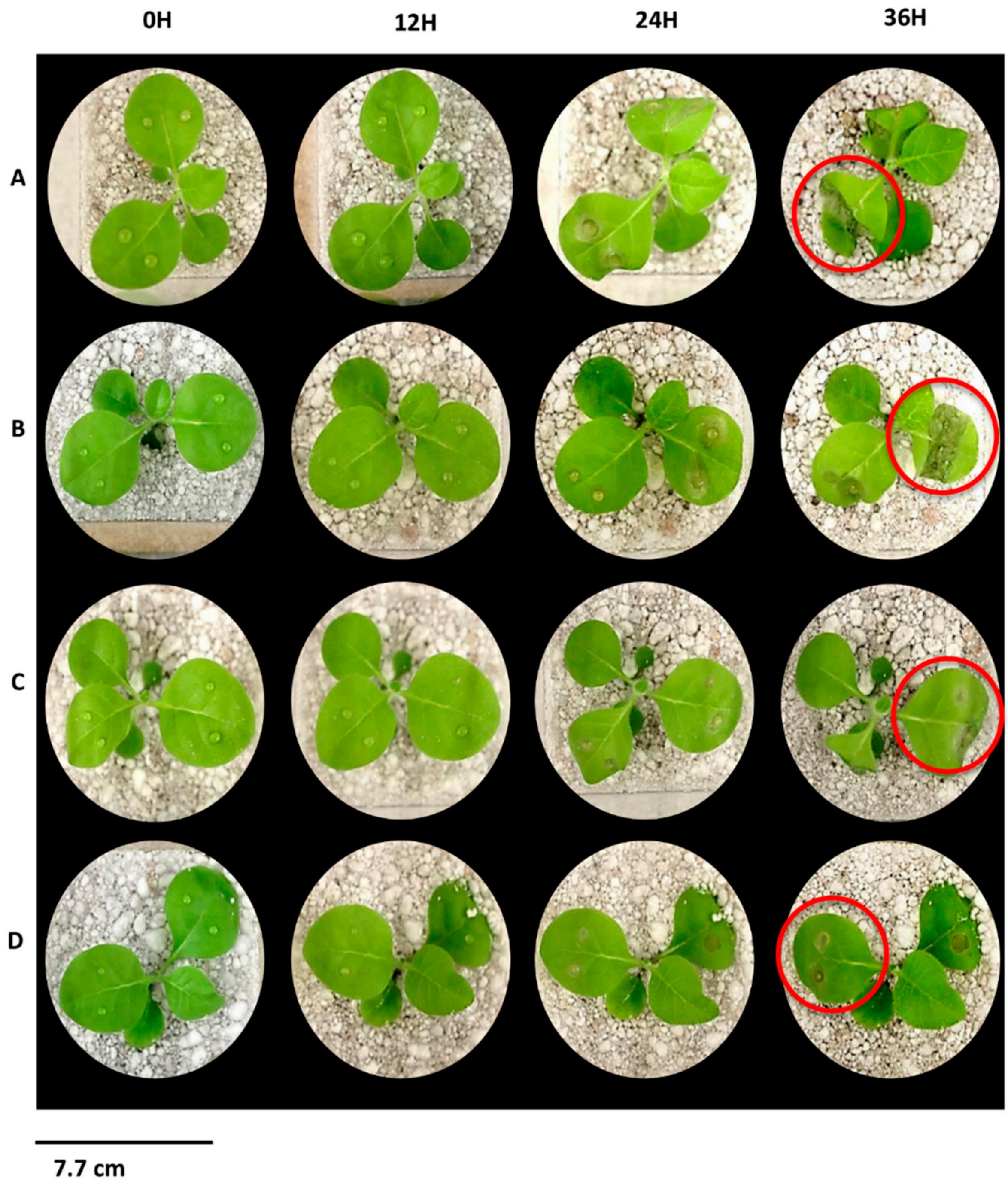

Figure S6. Disease symptom development of Nijmegen-1 treated tissue culture tobacco plants upon challenge with *B. cinerea*. Intact leaves were inoculated with a conidial suspension of B. cinerea and maintained at 22°C at 100% humidity. Treatments consisted of root drench applications of water for (A) control sets or Nijmegen-1 at concentrations (B) $1x10^{-7}$M, (C) $1x10^{-8}$M and (D) $1x10^{-9}$M. Images are representative of four biological repeats of two independent experiments. Disease development is indicated by the red circle.

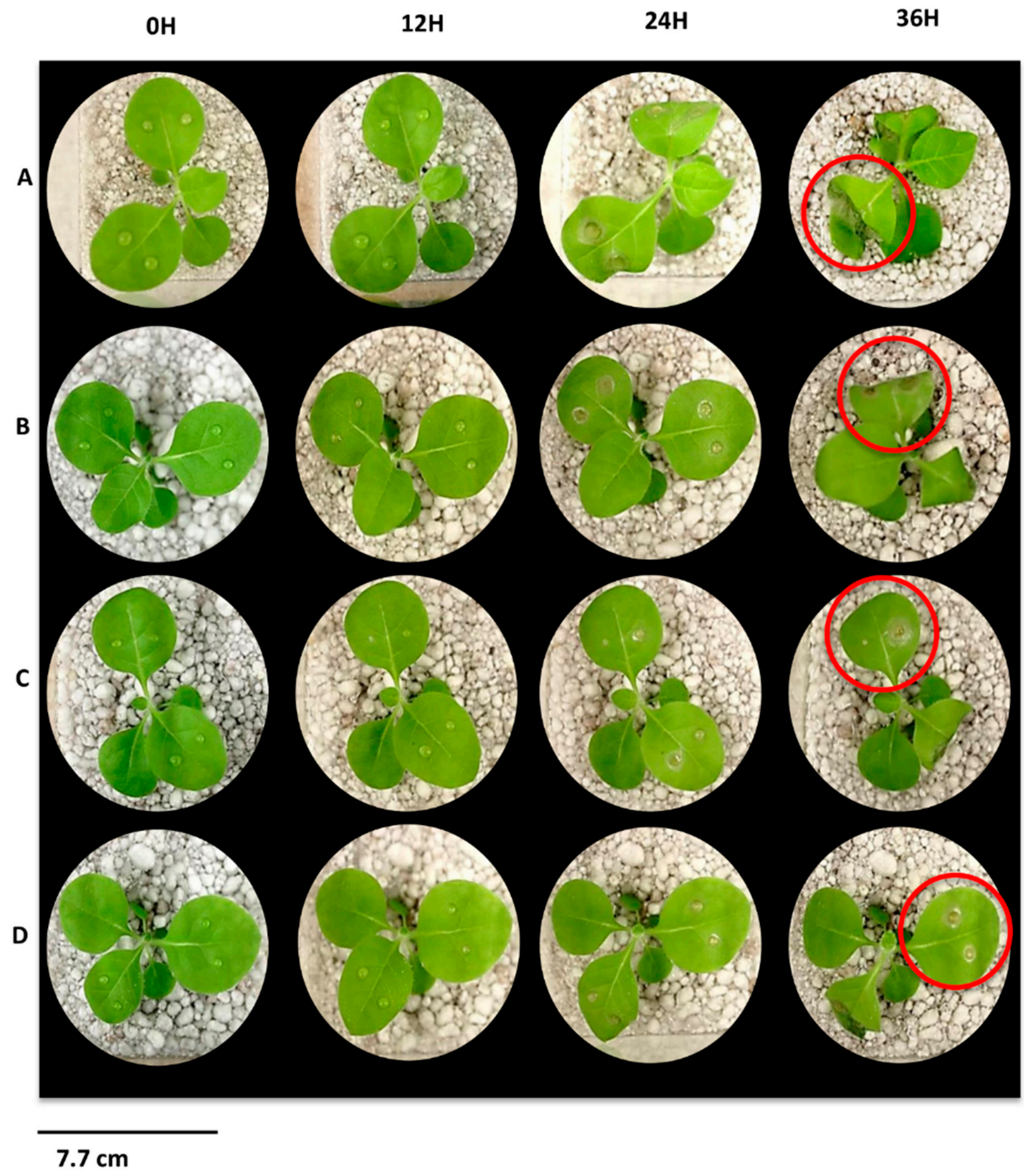

Figure S7. Disease symptom development of (±)-GR24 treated tissue culture tobacco plants upon challenge with *B. cinerea*. Intact leaves were inoculated with a conidial suspension of B. cinerea and maintained at 22°C at 100% humidity. Treatments consisted of root drench applications of water for (A) control sets or (±)-GR24 at concentrations (B) $1x10^{-7}$M, (C) $1x10^{-8}$M and (D) $1x10^{-9}$M. Images are representative of four biological repeats of two independent experiments. Disease development is indicated by the red circle.

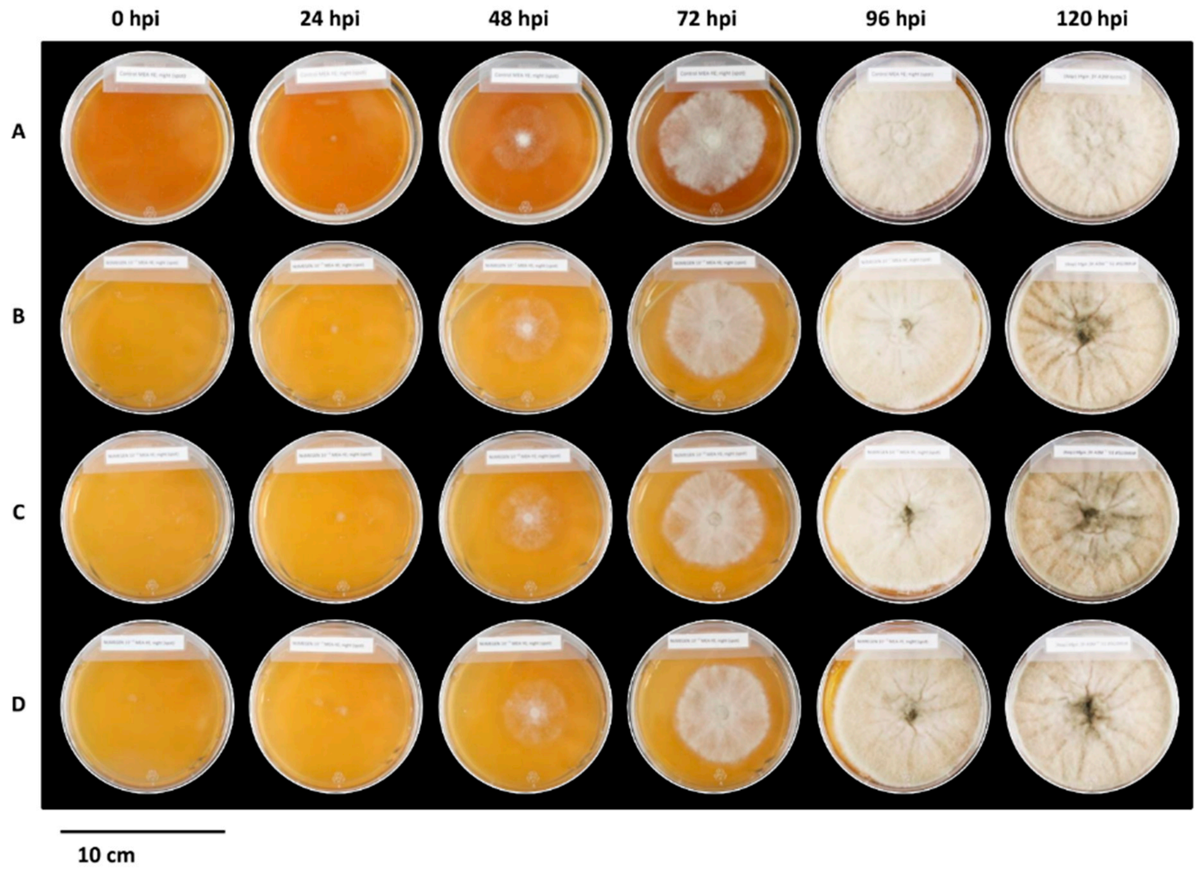

Figure S8. Effect of Nijmegen-1 on the radial growth of *B. cinerea*. The radial growth of *B. cinerea* was assayed on MEA-YE agar (A) or in the presence of various concentrations of Nijmegen-1at (B) $1x10^{-7}$M, (C) $1x10^{-8}$M and (D) $1x10^{-9}$M. Petri dishes were observed for 120 hours post inoculation (hpi). Images are representatives of four replicates of two independent experiments.

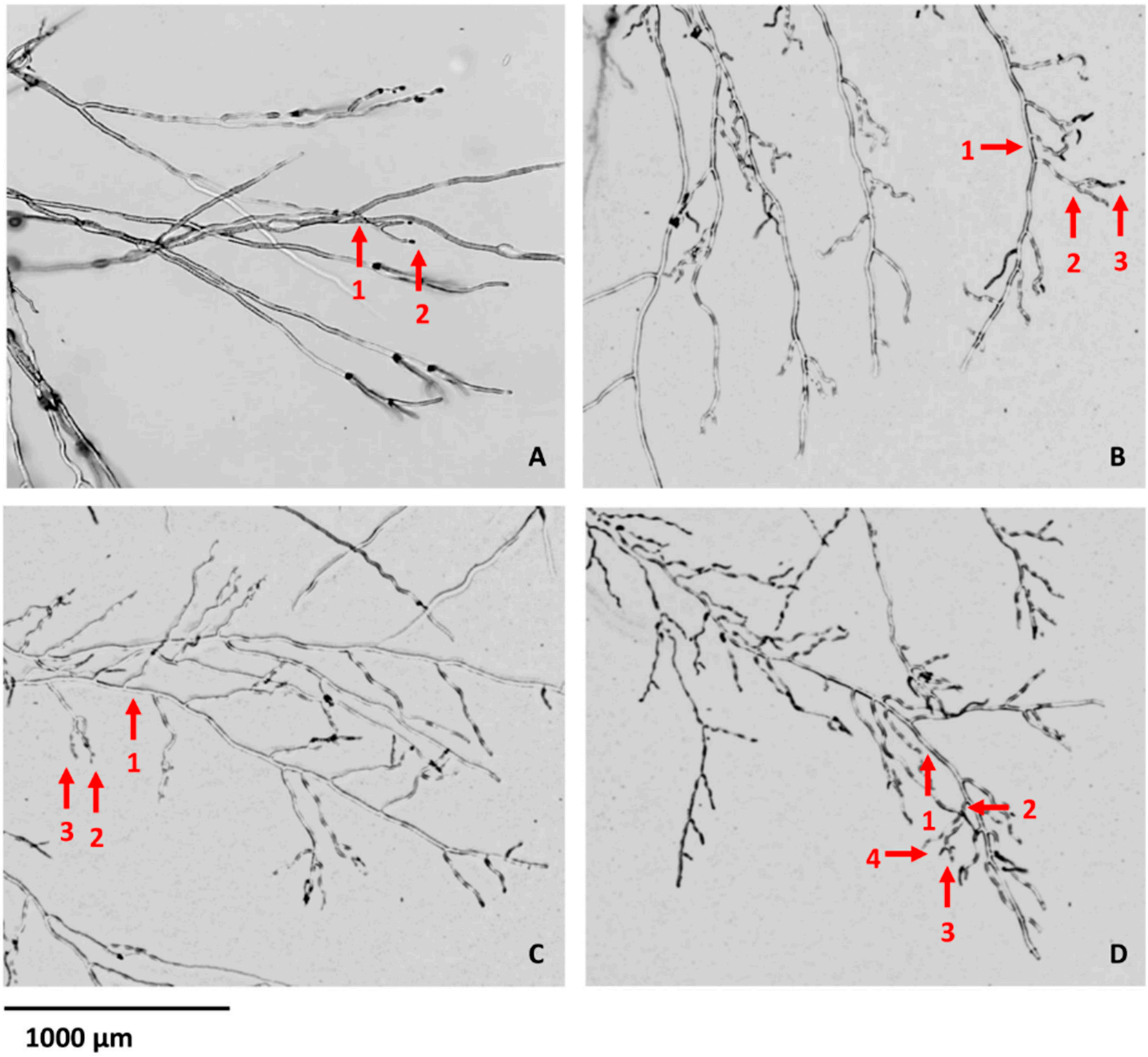

Figure S9. Effect of Nijmegen-1 on the hyphal branching structures of *B. cinerea*. The growth of *B. cinerea* was assayed on 1% water agar (A) or in the presence of various concentrations of Nijmegen-1at (B) $1x10^{-7}$M, (C) $1x10^{-8}$M and (D) $1x10^{-9}$M. Petri dishes were observed under a stereomicroscope after 5 days post inoculation. Images are representatives of four replicates of two independent experiments. The order of branching is indicated by red arrows and numbers.

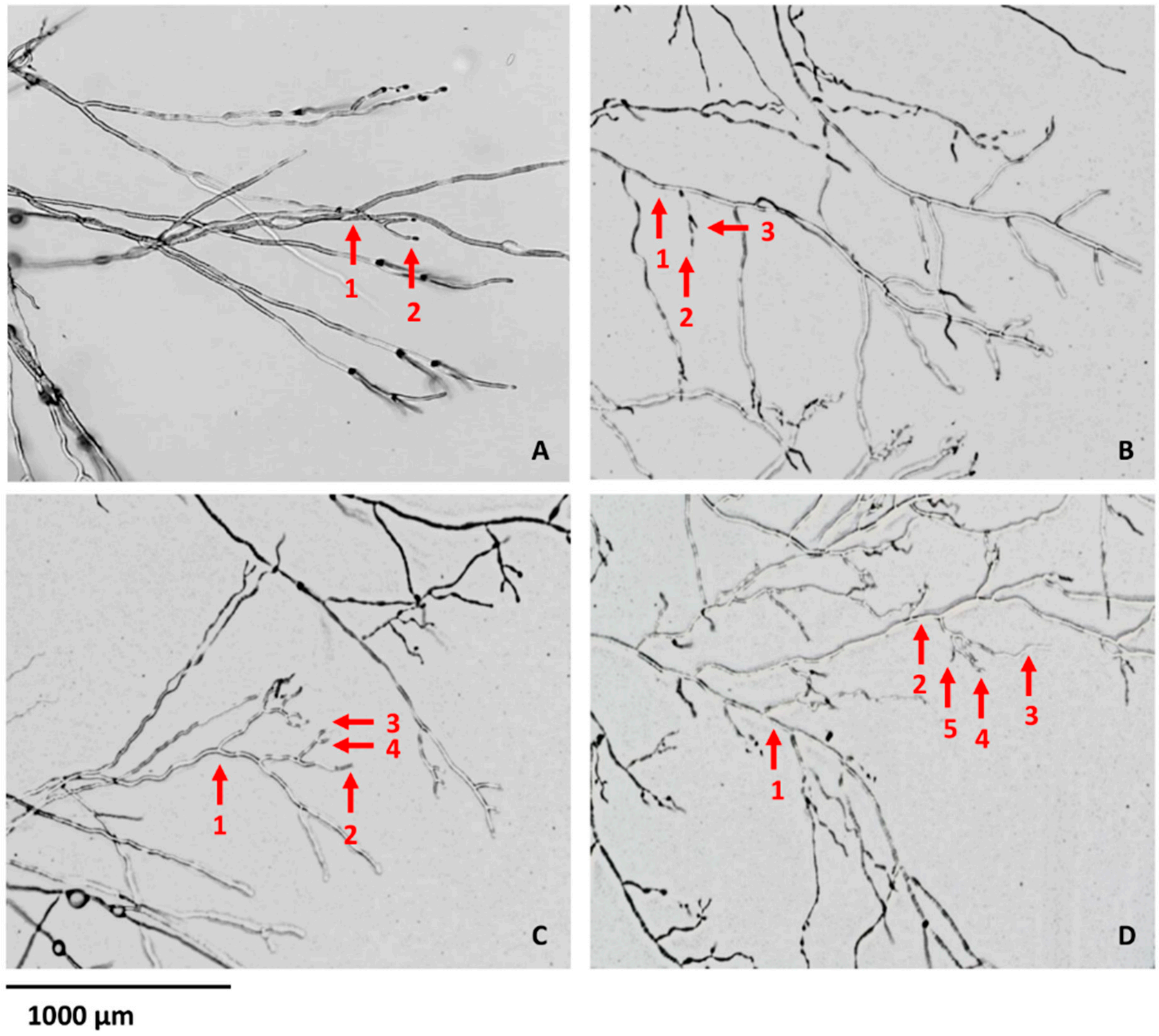

Figure S10. Effect of (±)-GR24 on the hyphal branching structures of *B. cinerea*. The growth of *B. cinerea* was assayed on 1% water agar (A) or in the presence of various concentrations of (±)-GR24 at (B) $1x10^{-7}$M, (C) $1x10^{-8}$M and (D) $1x10^{-9}$M. Petri dishes were observed under a stereomicroscope after 5 days post inoculation. Images are representatives of four replicates of two independent experiments. The order of branching is indicated by red arrows and numbers.
